# The involvement of CYP1A2 in biodegradation of dioxins in pigs

**DOI:** 10.1371/journal.pone.0267162

**Published:** 2022-05-26

**Authors:** Sylwia Swigonska, Tomasz Molcan, Anna Nynca, Renata E. Ciereszko

**Affiliations:** 1 Laboratory of Molecular Diagnostics, Faculty of Biology and Biotechnology, University of Warmia and Mazury in Olsztyn, Olsztyn, Poland; 2 Department of Bioinformatics, Institute of Biochemistry and Biophysics, Polish Academy of Sciences, Warsaw, Poland; 3 Department of Animal Anatomy and Physiology, Faculty of Biology and Biotechnology, University of Warmia and Mazury in Olsztyn, Olsztyn, Poland; Brooklyn College of the City University of New York, UNITED STATES

## Abstract

2,3,7,8-tetrachlorodibenzo-*p*-dioxin (TCDD) is one of the most harmful chemicals showing resistance to biodegradation. The majority of TCDD effects is mediated by the aryl hydrocarbon receptor (AhR) pathway. TCDD binding to AhR results in the activation of cytochrome P450 enzymes (CYP1A1, CYP1A2, CYP1B1) involved in dioxin biodegradation. The goal of the study was to explore the potential role of CYP1A2 in the metabolism of TCDD. We investigated a molecular structure of CYP1A2 and the binding selectivity and affinity between the pig CYP1A2 and: 1/ DiCDD or TCDD (dioxins differing in toxicity and biodegradability) or 2/ their selected metabolites. pCYP1A2 demonstrated higher affinity towards DiCDD and TCDD than other pCYP1 enzymes. All dioxin-pCYP1A2 complexes were found to be stabilized by hydrophobic interactions. The calculated distances between the heme oxygen and the dioxin carbon nearest to the oxygen, reflecting the hydroxylating potential of CYP1A2, were higher than in other pCYP1 enzymes. The distances between the heme iron and the nearest dioxin carbon exceeded 5 Å, a distance sufficient to allow the metabolites to leave the active site. However, the molecular dynamics simulations revealed that two access channels of CYP1A2 were closed upon binding the majority of the examined dioxins. Moreover, the binding of dioxin metabolites did not promote opening of channel S–an exit for hydroxylated products. It appears that the undesired changes in the behavior of access channels prevail over the hydroxylating potential of CYP1A2 towards TCDD and the favorable distances, ultimately trapping the metabolites at the enzyme’s active site.

## 1. Introduction

The global awareness concerning environmental pollutants is constantly growing. This is due to decades of industrialization which offered not only needed or desirable goods but also flooded us with waste by-products. Such by-products include polychlorinated dibenzo-*p*-dioxins (PCDDs, dioxins) that persistently contaminate our environment. The largest unintentional release of 2,3,7,8-tetrachlorodibenzo-*p*-dioxin (TCDD),the most toxic amongst 75 known dioxin congeners, occurs through waste incineration, metal production as well as petrol industry and wood combustion [1]. TCDD is considered to be one of the most harmful chemicals with long-lasting half-life (e.g., 8–10 years in humans). Exposure to TCDD results in numerous pathophysiological abnormalities such as chloracne, thymic atrophy and immune dysfunction, hepatic damage and steatosis, gastric epithelial hyperplasia, embryonic teratogenesis and cancer [2]. TCDD also affects male and female reproduction as well as endocrinology. It alters sexual behavior, decreases spermatogenesis, diminishes fertility, causes endometriosis, teratogenesis and abortion [3]. Moreover, the dioxin influences thyroid hormone metabolism as well as steroid hormone secretion [4, 5]. In pigs, TCDD was also found to affect the expression of genes involved in the regulation of granulosa cell cycle, proliferation and follicular atresia [6, 7].

Toxicity of dioxins is conditioned by the number and position of chlorine atoms present in its molecule. Dioxin congeners substituted in lateral positions with chlorine atoms, usually demonstrate high level of toxicity [8–10]. The extremely high toxicity of TCDD results from the occurence of chlorine atoms in all lateral positions and is accompanied by the highest resistance to biodegradation [9, 10] (. In contrast, 2,7-dichlorodibenzo-*p*-dioxin (DiCDD), less toxic than TCDD and containing unsubstituted lateral positions, appears to be effectively metabolized [11, 12]. TCDD’s high resistance to biodegradation results in its accumulation in adipose tissue and hence is, at least partially, responsible for its adverse effects exerted on living organisms [10, 13, 14]. It should be noted that both, toxicity and susceptibility of PCDDs to biodegradation are not only determined by a dioxin chemical structure but also are species-dependent [9, 10].

The majority of PCDD effects is mediated by the aryl hydrocarbon receptor (AhR) pathway. AhR is a highly conserved transcription factor activated by numerous exogenous ligands, including PCDDs, polyhalogenated dibenzofurans (PCDFs) and polyaromatic hydrocarbons (PAHs) [15]. The TCDD binding to AhR results in the translocation of the TCDD-AhR complex to the nucleus and dimerization with AhR nuclear translocator (ARNT). The TCDD-AhR-ARNT complex binds a dioxin response element (DRE) in promoter regions of TCDD target genes including those of phase I biotransformation enzymes, e.g., cytochrome P450(CYP1) family 1. CYP1A1, CYP1A2 and CYP1B1 are involved in the biodegradation of PCDDs and PAHs. In addition to contribution to phase 1 metabolism of drugs and xenobiotics, the enzymes play also a role in the development of many diseases including cancer [16].

The detoxification process starts with reactions of hydroxylation occurring in heme-containing active site of the enzyme [8, 15, 17, 18]. In our previous studies we analyzed the potential of pig CYP1A1 (pCYP1A; [12]) and CYP1B1 [19] to hydroxylate dioxins differing in toxicity and biodegradability. We have reported that TCDD, but not DiCDD, was not effectively metabolized by pCYP1A1 in pigs mainly due to the specific behavior of substrate channels leading into the active site of the enzyme. *In silico* analysis demonstrated that TCDD, upon its binding by CYP1A1, is not able to leave the enzyme’s active site because of the closure of the access channels [12]. Similar analysis performed for pig CYP1B1 demonstrated a smaller distance between heme oxygen and the nearest TCDD carbon atom, reflecting the higher hydroxylating potential of pig CYP1B1 than CYP1A1 [19]. Additionally, compared to CYP1A1, TCDD binding to the CYP1B1 active site results in a higher availability of access channel S–considered to be an exit channel for hydroxylation products [19, 20]. On the other hand, the smaller volume of the CYP1B1 active site may hinder the mobility of TCDD molecule. This fact together with the long half-life of TCDD associated with ineffective biodegradation suggests that there are other factors inhibiting hydroxylation of the dioxin. However, the exact mechanism responsible for this inhibition within both CYPs is still not recognized. Therefore, CYP1A2, the last member of the CYP1 family, directly involved in the metabolism of xenobiotics [21] was a natural candidate for testing its ability to metabolize dioxins. Different dynamics of the loop-like structures forming the substrate binding cavity of a particular CYP1 enzyme additionally justify the idea to explore the dioxin-CYP1A2 complexes. Moreover, contrary to CYP1A1 and CYP1B1, ubiquitously distributed in many animal tissues, the presence of CYP1A2 is limited mainly to the liver [22, 23] which may also affect functioning of the enzyme.

It is commonly known that, contrary to other dioxins, biodegradation of TCDD is extremely slow and inefficient. However, a few *in vitro* and *in vivo* studies demonstrated the generation of several mono-hydroxylated TCDD metabolites in humans, rats and dogs [9, 11, 14, 24, 25]. Therefore, in addition to TCDD and DiCDD, some selected primary TCDD metabolites as well as their molecular interactions with CYP1A2 were examined in the current study. The main goal of the present study was to explore the potential role of CYP1A2 in the metabolism of PCDDs in the pig. To achieve this goal, we investigated *in silico* a molecular structure of CYP1A2 as well as the binding selectivity and affinity between the pig CYP1A2 and DiCDD or TCDD, the two dioxins differing in toxicity and biodegradability. Similar analysis was also performed for four selected dioxin metabolites. In addition, the accessibility of CYP1A2 access channels upon binding of dioxins and their metabolites was investigated.

## 2. Results

### 2.1. In silico model of the pig CYP1A2 catalytic domain

**Fig 1:**
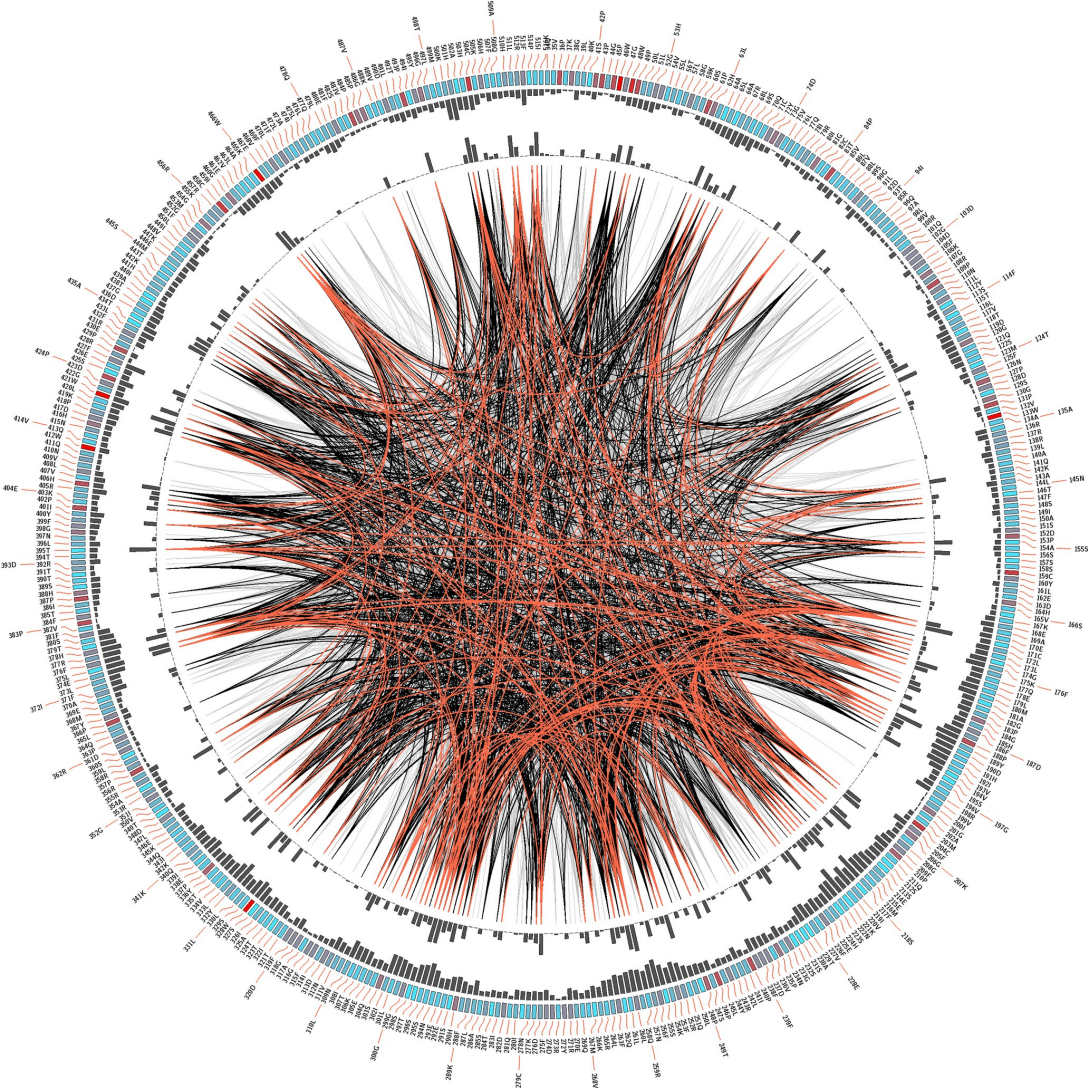
CIRCOS analysis of CYP1A2 i.e., interspecies alignment of the enzyme sequence.

**Fig 2:**
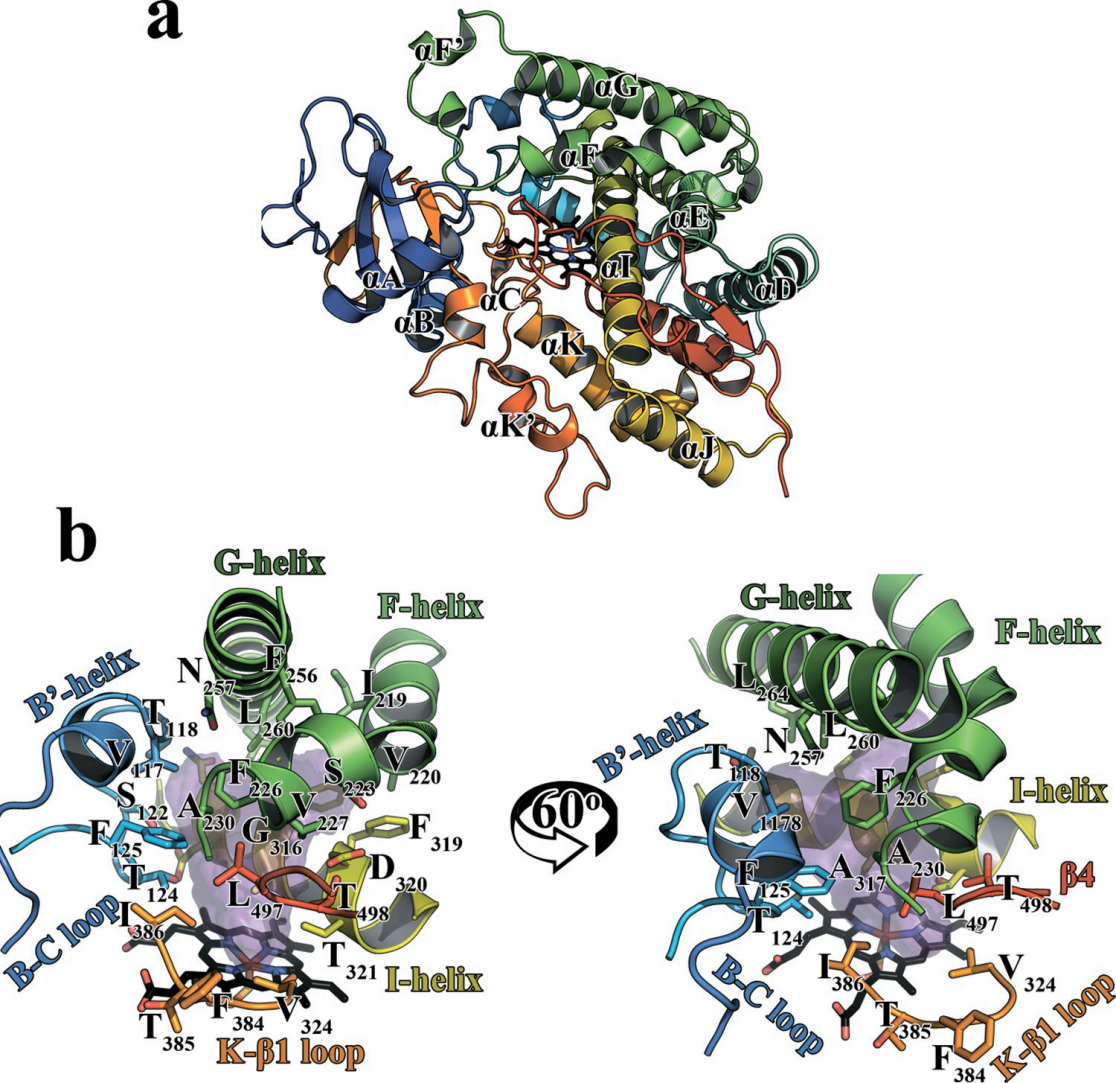
The overall three-dimensional structure of pig CYP1A2 (pCYP1A2) catalytic domain **(a).** The N-terminal end of the amino acid chain is marked in dark blue, the C-terminal end is marked in red; different letters and colors (A–L) depict 12 α-helices of the protein. Topology of the pCYP1A2 active site (b). The active site of pCYP1A2 is presented as a violet area. Heme molecule of the active site is shown in black. Residues bordering the active site are depicted as color sticks. Helices (G, I, B′, F) and loops (B-C, K-β1) surrounding the active site are shown in different colors. A characteristic three-aa break (221–223) in helix F, typical for mammalian pCYP1A2 enzymes, is visible in the aa sequence.

The similarity level of the CYP1A2 amino acid (aa) sequence between the pig and other species ranged from71.40% (*Mus pahari*) to 86.05% (*Camelus ferus*) (S1 Table, Fig 1). It was also found that aa sequence of pCYP1A2 shared 81.40% homology with human CYP1A2 (S1 Fig). The tertiary structure of the pCYP1A2 catalytic domain is shown in Fig 2A. The pCYP1A2 protein is composed of 12 canonical α-helices (A-L) and six β-sheets (β1- β6). In addition, the spatial structure of pCYP1A2 includes three short helices (B′, F′ and K′) anchored in the membrane. These helices are frequently involved in the formation of substrate channels within the enzyme active site. A characteristic three-aa break (221–223) in helix F, typical for mammalian pCYP1A2 enzymes, was also found in the pig (S1 Fig). The PROCHECK evaluation of the final pCYP1A2 model revealed that 94.2% of its residues were located in the most favorable region (red) of Ramachandran plot (S2 Fig). The Z-score provided by ProSA-web indicated the overall pCYP1A2 model quality as -9.34, while the overall model quality for human CYP1A2 was estimated at-10.28.Moreover, the model evaluation performed by VERIFY3D indicated that 94.0%of residues produced scores higher than 0.2. The achieved results confirm that the generated homology-based model of pCYP1A2 protein is characterized by high quality parameters and therefore highly reliable.

We have also shown that the deeply buried active site of the pCYP1A2 was bordered by K-β1 and B-C loops, G, I, B′ and F helix regions and β4 sheet (Fig 2). The volume of the active site was calculated to be 436.98 Å^3^.

The pig CYP1A2 protein was used as reference. Letter codes in the first (outer) circle indicate the alignment position and the amino acid code of the reference sequence. The colored square boxes of the second circle indicate the MSA position conservation (highly conserved positions are presented in red, while less conserved in blue). The third circle shows the cumulative mutual information as histograms, facing outwards. In the center of the circle lines can be observed, that connect pairs of positions with mutual information greater than 6.5. Red edges represent the top 5%, black lines represent points scoring between 95–70%, and gray edges account for the remaining 70%.

### 2.2. Docking and molecular dynamics simulation of the PCDD-porcine CYP1A2 complexes

Molecular docking confirmed the high reliability of the constructed pCYP1A2 model. All examined dioxins (DiCDD, TCDD and four metabolites) assumed the same orientation within the CYP1A2 active site (S3 Fig). The amino acids involved in the stabilization of a particular dioxin molecule within the enzyme active site are presented in Table 1. The dioxins were found to be stabilized by hydrophobic interactions including π-stacking interactions between a dioxin molecule and the residues of the pCYP1A2.

**Table 1:** The pCYP1A2 residues involved in molecular interactions with the examined dioxins.

Compounds	After molecular docking		AfterMD simulations	
	Hydrophobic interactions	π-π interactions	Hydrophobic interactions	π-π interactions
DiCDD	**L**_**260**_ **G**_**316**_ **A**_**317**_ T_321_ V_382_ **I**_**386**_	F_125_ **F**_**226**_	T_124_ **G**_**316**_ **A**_**317**_ **T**_**321**_ V_382_ I_386_ **L**_**497**_	F_125_ **F**_**226**_
3OH-DiCDD	T_124_ S_223_ V_227_ **L**_**260**_ **G**_**316**_ **A**_**317**_ D_320_ T_321_ **I**_**386**_	**F**_**226**_ F_256_	N_257_ L_260_ N_312_ **G**_**316**_ **A**_**317**_ **T**_**321**_ **L**_**497**_	F_125_ **F**_**226**_
TCDD	T_124_ S_223_ **L**_**260**_ **G**_**316**_ **A**_**317**_ D_320_ **I**_**386**_	**F**_**226**_ F_256_	**G**_**316**_ **A**_**317**_ D_320_ **T**_**321**_ V_382_ I_386_ **L**_**497**_	F_125_ **F**_**226**_ F_319_
8OH-TriCDD	**L**_**260**_ **G**_**316**_ **A**_**317**_ T_321_ V_382_ **I**_**386**_ T_498_	F_125_ **F**_**226**_ F_256_	L_260_ **G**_**316**_ **A**_**317**_ **T**_**321**_ V_382_ I_386_ **L**_**497**_	F_125_ **F**_**226**_ F_319_
1OH-TCDD	T_124_ S_223_ V_227_ **L**_**260**_ **G**_**316**_ **A**_**317**_ D_320_ T_321_ V_382_ **I**_**386**_ T_498_	**F**_**226**_ F_256_	T_118_ T_124_ L_260_ **G**_**316**_ **A**_**317**_ **T**_**321**_ V_382_ I_386_ **L**_**497**_	F_125_ **F**_**226**_ F_319_
2OH-TCDD	T_124_ **L**_**260**_ N_312_ **G**_**316**_ **A**_**317**_ T_321_ V_382_ **I**_**386**_ T_498_	F_125_ **F**_**226**_ F_256_	T_124_ S_223_ G_227_ L_260_ **G**_**316**_ **A**_**317**_ **T**_**321**_ V_382_ I_386_ **L**_**497**_ T_498_	**F**_**226**_ F_256_ F_319_

Bold letters indicate amino acids shared between dioxins and metabolites, red letters indicate amino acids shared between DiCDD and TCDD

**Fig 3:**
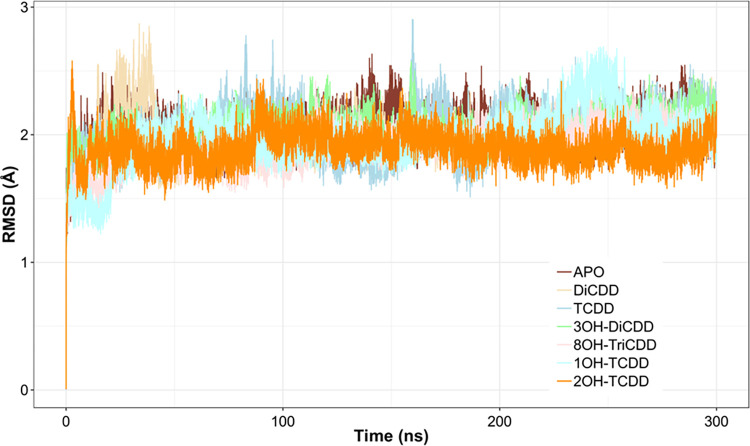
The root-mean-square deviation (RMSD) values collected during MD simulations of the examined dioxin-pCYP1A2 complexes (100–300 ns); APO–substrate-free form of pCYP1A2.

**Fig 4:**
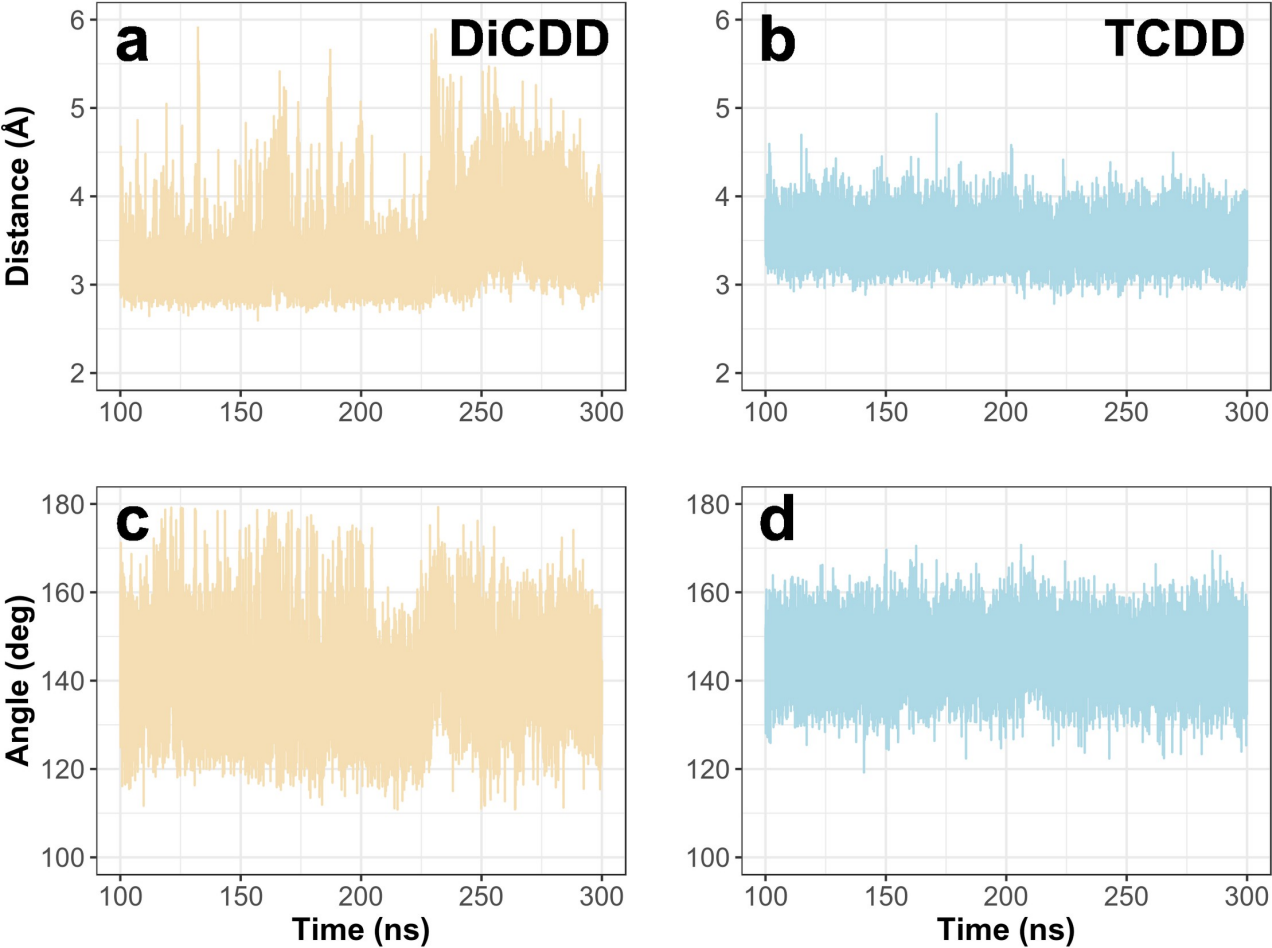
Time-dependent changes in the distance between the pCYP1A2 heme oxygen and the dioxin carbon atom that is nearest to the oxygen (a, b) and in the angle between the pCYP1A2 heme iron, oxygen and the dioxin carbon atom that is nearest to the oxygen (c, d).

**Fig 5:**
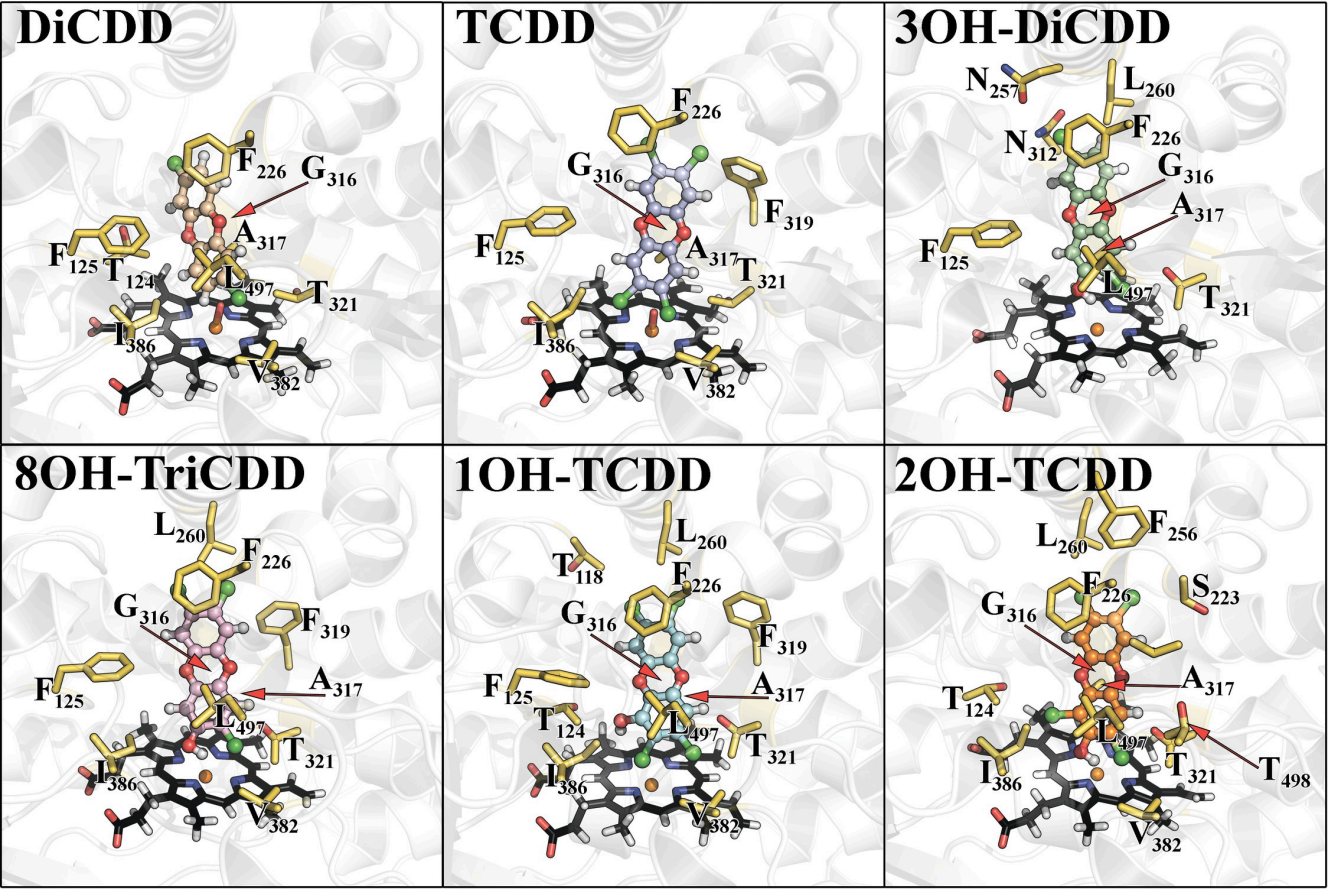
The visualization of a dioxin position in the active site of pCYP1A2duringmolecular dynamics simulations. Side chains of pCYP1A2 amino acids interacting with the examined dioxin are depicted in yellow; heme is black; DiCDD is beige, TCDD is lavender, 3OH-DiCDD is green, 8OH-TriCDD is pink, 1OH-TCDD is pale willow-green and 2OH-TCDD as orange.

During MD simulation the complexes reached equilibrium after the first 100 ns of the simulation (Fig 3, S2 Table). To examine the potential of the pCYP1A2 to hydroxylate dioxin congeners, the distance between the heme oxygen atom and the dioxin carbon atom nearest to the oxygen was calculated. The calculated distances oscillated around 3.36 ± 0.42Å for DiCDD and 3.50 ± 0.22 Å for TCDD (Fig 4A and 4B). In addition, the angle between the heme iron, oxygen and the dioxin carbon atom that is nearest to the oxygen was also measured. This angle oscillated around 138.42 ± 0.06° and 144.92 ± 0.04° for DiCDD and TCDD, respectively (Fig 4C and 4D). The visualization of a particular dioxin position within the active site of pCYP1A2 during MD simulation is presented in Fig 5. All examined congeners were located in the pCYP1A2 active site directly above the heme. Dioxin molecules were found to be stabilized in the active site solely by hydrophobic interactions (Table 1, Fig 5). Each dioxin was stabilized primarily by G_316_, A_317_, T_321_, V_382_, I_386_and L_497_. Additionally, π-π interactions formed by F_125_and F_226_ were also involved in the stabilization of each dioxin within the pCYP1A2 active site.

**Fig 6:**
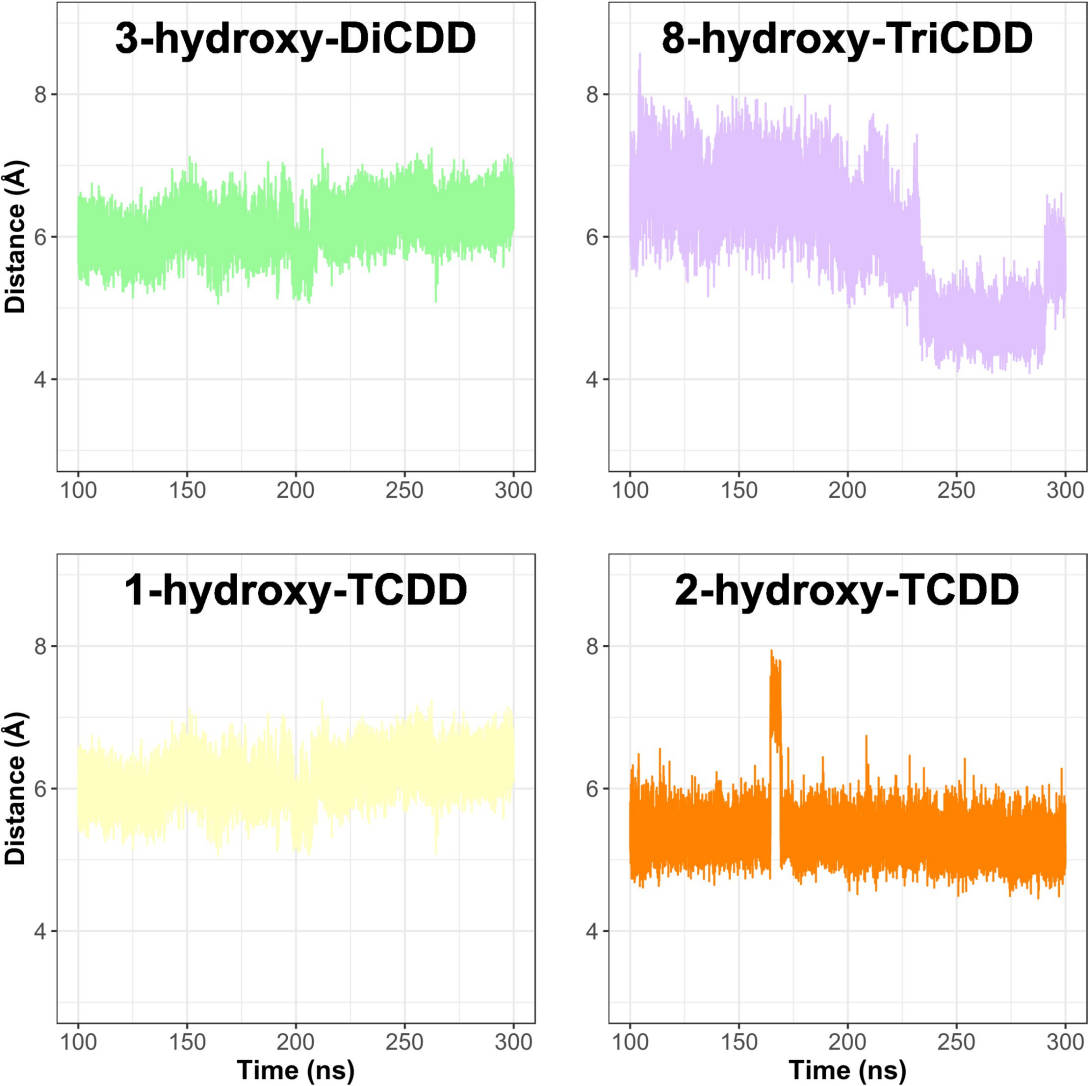
Time-dependent changes in the distance between the pCYP1A2 heme iron and the dioxin metabolite carbon atom that is nearest to the iron. 3OH-DiCDD– 3-hydroxy-2,7-dichlorodibenzo-*p*-dioxin, 8OH-TriCDD– 8-hydroxy-2,3,7-trichlorodibenzo-*p*-dioxin, 1OH-TCDD– 1-hydroxy-2,3,7,8-tetrachlorodibenzo-*p*-dioxin, 2OH-TCDD– 2-hydroxy-1,3,7,8-tetrachlorodibenzo-*p*-dioxin.

The distance between the heme iron atom and the dioxin metabolite carbon atom that is nearest to the iron was calculated to assess the potential of a hydroxylated metabolite to leave the pCYP1A2 active site. The calculated distances oscillated around 5.36 ± 0.35 Å for 3OH-DiCDD, 5.71 ± 0.24 Å for 8OH-TriCDD, 6.13 ± 0.31 Å for 1OH-TCDD and 5.35± 0.37 Å for 2OH-TCDD (Fig 6). Each metabolite was stabilized primarily by G_316_ A_317_ T_321_and L_497_. Additionally, π-π interactions formed by F_226_ were also involved in the stabilization of each metabolite in the pCYP1A2 active site (Table 1, Fig 5).

### 2.3. Molecular dynamics simulation of PCDD-human CYP1A2 complexes

Similar to pCYP1A2, the distance between the heme oxygen atom and the dioxin carbon atom that is nearest to the oxygen was also calculated for human CYP1A2. The calculated distances oscillated around 4.15 ± 0.54 Å for DiCDD and 3.85 ± 0.34Å for TCDD (S4A and S4B Fig). In addition, the angle between the heme iron, oxygen and the dioxin carbon atom that is nearest to the oxygen oscillated around 145.45 ± 0.1° and 150.96 ± 0.9° for DiCDD and TCDD, respectively (S4C and S4D Fig).

### 2.4. Calculated binding affinity of the PCDD-pCYP1A2 complexes after MD simulations and thermodynamic integration

The binding free energy of the TCDD-pCYP1A2 complex (-13.671±0.020 kcal·mol^−1^) was lower than that of the DiCDD-pCYP1A2 complex (-12.778±0.026 kcal·mol^−1^) (Table 2). The binding free energy for DiCDD, in turn, was lower in comparison to that for 3OH-DiCDD (-9.697±0.053 kcal·mol^−1^). Similarly, 8OH-TriCDD (-12.007 ± 0.032 kcal·mol^−1^) showed a slightly lower affinity to the pCYP1A2 active site than TCDD. In contrast, the two other TCDD metabolites *i*.*e*., 1OH-TCDD (-24.415±0.050 kcal·mol^−1^) and 2OH-TCDD (-15.324±0.043 kcal·mol^−1^) showed higher affinity to the pCYP1A2 active site than TCDD. The pCYP1A2 demonstrated higher affinity towards DiCDD and TCDD in comparison to other members of pCYP1 enzymes [12, 19] (S3 Table).

**Table 2:** The absolute binding free energy (kcal·mol^-1^) calculated through MD simulations and thermodynamic integration for the dioxin-pCYP1A2 complexes.

Energy component	DiCDD	3OH-DiCDD	TCDD	8OH-TriCDD	1OH-TCDD	2OH-TCDD
${\Delta G}_{elec+vdw+rest}^{prot}$	-22.369±0.022	-21.939±0.051	-23.335±0.013	-23.825±0.027	-37.629±0.045	-28.110±0.040
${\Delta G}_{elec+vdw}^{solv}$	2.611±0.014	5.077±0.016	2.497±0.016	4.996±0.017	6.423±0.021	5.822±0.016
${\Delta G}_{rest}^{solv}$	6.980	7.165	6.894	6.936	6.791	6.964
${\Delta G}_{binding}^{0}$	**-12.778±0.026**	**-9.697±0.053**	**-13.671±0.020**	**-12.007±0.032**	**-24.415±0.050**	**-15.324±0.043**

${\Delta G}_{elec+vdw+rest}^{prot}$–ligand decoupling from complex; ${\Delta G}_{elec+vdw}^{solv}$–ligand decoupling from solution; ${\Delta G}_{rest}^{solv}$–ligand restraints added to decoupled ligand; ${\Delta G}_{binding}^{0}$–absolute binding free energy, ${\Delta G}_{binding}^{o}={\Delta G}_{elec+vdw+rest}^{prot}+{\Delta G}_{elec+vdw}^{solv}+{\Delta G}_{rest}^{solv}$

DiCDD– 2,7-dichlorodibenzo-*p*-dioxin, TCDD– 2,3,7,8-tetrachlorodibenzo-*p*-dioxin, 3OH-DiCDD– 2,7-dichloro-3-hydroxy-dibenzo-*p*-dioxin

8OH-TriCDD– 2,3,7-trichloro-8-hydroxy-dibenzo-*p*-dioxin, 1OH-TCDD– 2,3,7,8-tetrachloro-1-hydroxy-dibenzo-*p*-dioxin, 2OH-TCDD– 1,3,7,8-tetrachloro-2-hydroxy-dibenzo-*p*-dioxin

In addition, the binding free energy of each residue forming all the examined dioxin-pCYP1A2 complexes was analyzed to estimate the influence of particular residues on the dioxin-enzyme binding. The amino acids with the strongest impact on the formation of the dioxin-pCYP1A2 complexes are listed in Table 3 and visualized in Fig 5. The complexes were found to be stabilized by hydrophobic interactions including π-stacking interactions (F_125_ and F_226_) between a dioxin and pCYP1A2.

**Table 3:** The relative binding free energy (ΔΔG;kcal·mol^-1^) calculated for individual amino acids stabilizing the dioxin-pCYP1A2 complexes.

Dioxin	F_125_A	F_226_A	G_316_A	A_317_G	T_321_A	V_382_A	I_386_A	L_497_A
**DiCDD**	1.527±0.033	2.202±0.015	0.615±0.003	0.990±0.101	1.122±0.012	0.897±0.008	1.131±0.014	2.910±0.017
**3OH-DiCDD**	–	-0.344±0.027	-0.248±0.004	1.303±0.004	1.776±0.014	–	–	2.960±0.034
**TCDD**	0.807±0.031	2.010±0.012	0.091±0.004	0.471±0.004	0.960±0.007	-0.040±0.007	0.388±0.009	3.508±0.013
**8OH-TriCDD**	–	-0.322±0.021	-0.386±0.003	0.745±0.004	0.270±0.07	–	–	4.443±0.031
**1OH-TCDD**	–	-0.039±0.024	0.747±0.004	0.142±0.004	1.761±0.08	–	–	2.612±0.032
**2OH-TCDD**	–	2.416±0.024	1.530±0.004	1.130±0.003	-0.087±0.009	–	–	3.354±0.024

DiCDD– 2,7-dichlorodibenzo-*p*-dioxin, TCDD– 2,3,7,8-tetrachlorodibenzo-*p*-dioxin, 3OH-DiCDD– 2,7-dichloro-3-hydroxy-dibenzo-*p*-dioxin

8OH-TriCDD– 2,3,7-trichloro-8-hydroxy-dibenzo-*p*-dioxin, 1OH-TCDD– 2,3,7,8-tetrachloro-1-hydroxy-dibenzo-*p*-dioxin, 2OH-TCDD– 1,3,7,8-tetrachloro-2-hydroxy-dibenzo-*p*-dioxin

The value for wild type of the pCYP1A2 bound DiCDD or TCDD was used as a reference (ΔG = 0)

### 2.5. Access channels of pig CYP1A2

**Fig 7:**
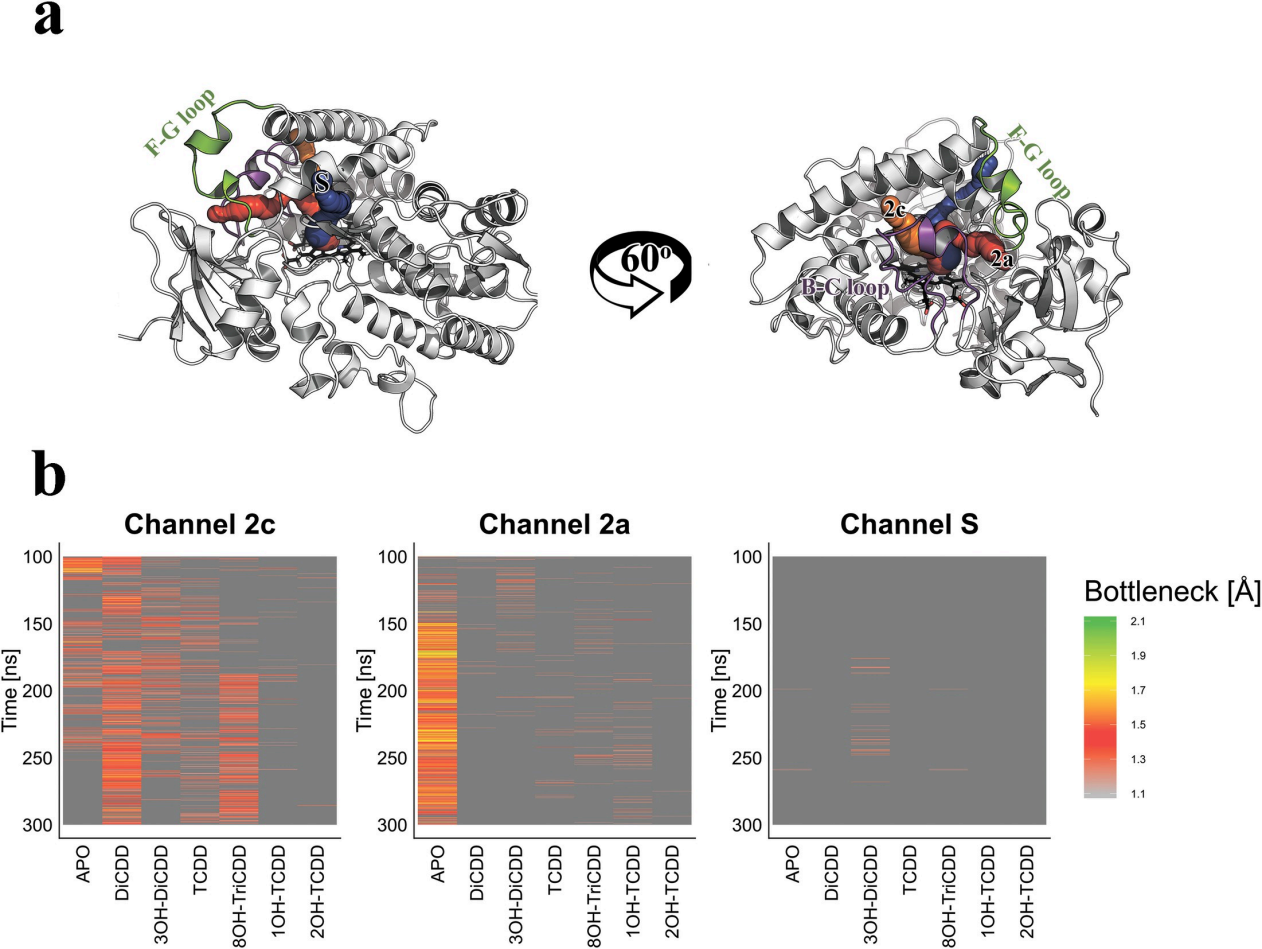
The access channels of the pCYP1A2 molecule shown in two different spatial enzyme orientations (a). Channel S is depicted in blue, channel 2c in orange, channel 2a in red and the heme molecule is shown in black. The F-G (green) and B-C (violet) loops are of particular importance for the state of the channel (open *vs*. closed). Dioxin-induced changes in the availability of the access channels identified within the pCYP1A2 molecule (b); Apo–substrate free form of pCYP1A2.

The specific interactions of a particular dioxin with pCYP1A2 affected the opening status (open vs. closed) of the substrate channels leading to the active site of the enzyme (Fig 7). To assess the potential of pCYP1A2 to hydroxylate a dioxin, the availability of access channels were analyzed in depth. The MD simulations revealed the presence of two channels (channel 2c and 2a) that were most frequently activated (opened) upon binding the examined dioxins (Fig 7A). Therefore, the subsequent analysis was performed only with regard to these two channels (Table 4). The accessibility of the substrate channels during the simulation time is shown in Fig 7B. In substrate-free pCYP1A2, channels 2c and 2a were open for 25.35% and 74.42% of the simulation time, respectively. Compared to the APO form of the enzyme, the presence of DiCDD or 8OH-TriCDD within the pCYP1A2 active site promoted the opening of the channel 2c for 63.30% or 35.68%, respectively. Furthermore, the binding of TCDD or its two metabolites i.e., 1OH-TCDD and 2OH-TCDD to the active site of the pCYP1A2 resulted in a closure of channel 2c (Table 4). The binding of all examined dioxins resulted in a closure of channel 2a. The presence of dioxin metabolites within the enzyme active site did not promote opening of channel S (Fig 7B).

**Table 4:** The features of the two most accessible access channels of pCYP1A2.

Name	Availability of the channel^a^	Mean bottleneck radius (Å)	Maximal bottleneck radius (Å)	Mean pathway length (Å)
**Channel 2c**				
APO	25.35%	1.35±0.13	1.87	23.95
DiCDD	63.30%	1.35±0.12	1.85	23.67
3OH-DiCDD	26.80%	1.31±0.09	1.70	22.71
TCDD	19.60%	1.28±0.07	1.57	23.07
8OH-TriCDD	35.68%	1.31±0.08	1.64	24.93
1OH-TCDD	2.03%	1.28±0.08	1.55	26.25
2OH-TCDD	1.00%	1.26±0.07	1.50	26.47
**Channel 2a**				
APO	74.42%	1.48±0.15	2.07	23.22
DiCDD	0.13%	1.24±0.03	1.32	30.37
3OH-DiCDD	5.45%	1.27±0.06	1.53	28.45
TCDD	1.95%	1.25±0.05	1.44	29.18
8OH-TriCDD	4.35%	1.25±0.05	1.44	28.32
1OH-TCDD	5.08%	1.26±0.06	1.48	25.38
2OH-TCDD	0.62%	1.23±0.03	1.32	26.58

## 3. Discussion

The members of the CYP1 family (CYP1A1, CYP1A2 and CYP1B1) are directly involved in biotransformation of many important endogenous and exogenous substances including steroid hormones, drugs or dioxins [21]. Previously, we have examined the potential of pig CYP1A1 [12] or CYP1B1 [19] to hydroxylate dioxins which differ in their toxicity and biodegradability. In the current study, we have investigated *in silico* the binding affinity and selectivity between pig CYP1A2 and DiCDD–a less toxic and easily biodegraded dioxin or TCDD–a dioxin that is strongly toxic and highly resistant to biodegradation. These molecular interactions were also analyzed between pCYP1A2 and four selected dioxin metabolites: 3OH-DiCDD, 1OH-TCDD, 2OH-TCDD and 8OH-TriCDD [11, 14, 26]. Moreover, we have studied the availability of access channels within the pCYP1A2 molecule after dioxin binding to analyze the potential routes of the entrance of the ligands and the exit of the hydroxylated products.

The concept of CYP-mediated metabolism of TCDD relies entirely upon the research reporting the formation of the dioxin metabolites in human and animal tissues [14, 24, 27] and/or the presence of catalytic activity of CYP1 enzymes towards TCDD [11, 25]. The presence of TCDD metabolites–as well as those of other dioxins–was detected in human faeces, blood serum and urine as well as in body fluids of mice and dogs [14, 24, 27]. Moreover, despite the documented formation of TCDD metabolites, the reason for slow and non-efficient degradation of this dioxin is still unknown.

The CYP-mediated hydroxylation process of dioxins is initiated by an effective binding of a specific substrate. The binding free energy reflects the binding affinity–the lower the energy, the higher the affinity. The results of the current study demonstrated that binding free energy of the TCDD-pCYP1A2 complex was lower than that of the DiCDD-pCYP1A2 complex, suggesting that TCDD is held stronger within the active site of CYP1A2. Several studies demonstrated a growing ligand binding affinity accompanying successive substituting the ligand molecule with chlorine atoms [28–30].

The binding specificity data demonstrated that the examined dioxins were stabilized within the CYP1A2 active site mostly *via* hydrophobic interactions. Among these, the π-stacking interactions, formed by F_115_or F_226_, seem to play an important role in the stabilization of a dioxin in the enzyme catalytic site. In addition, it was demonstrated that all examined dioxins assumed the same plane orientation within the CYP1A2 active site. The incorporation of hydroxyl group to the substrate is the first step of dioxin biodegradation catalyzed by CYP1 enzymes [9, 11]. The efficiency of the hydroxylation depends on the distance between the oxygen atom of the CYP1A2’s heme and the nearest carbon atom of the dioxin [31]. (It was suggested by Lonsdale et al. [32] that the distance between the CpdI oxygen and the ligand should range within 3-5Å to enable hydroxylation to occur. The distances between the oxygen atom of the enzyme’s heme and a particular dioxin (DiCDD or TCDD) oscillated around 3–3.5Å. Such a small distance implies the potential of being hydroxylated by pCYP1A2. Similar results were obtained for hCYP1A2 (S4 Fig). On the other hand, the values of measured angles between CYP1A2 and DiCDD or TCDD exceeded the values described by Lonsdale et al. [32] as being effective in incorporating a hydroxyl group into the aromatic ring. Moreover, apart from the enzyme’s hydroxylating potential, the successful biodegradation of dioxins also requires an effective evacuation of the polar product from the active site [20, 33]. We found that access channels S and 2a remained closed during the MD simulation of the two dioxins, while the 2c channel was opened only by DiCDD. The results suggest that after binding, both dioxins remain locked within the CYP1A2 active site, making hydroxylation impossible to proceed and also blocking the enzyme for other ligands. Moreover, the sequestration of TCDD within the pCYP1A2 may foreclose the dioxin molecule from being detoxified by other enzymes, which may be partially responsible for high toxicity of TCDD [24].

Analysis of binding free energy revealed that pCYP1A2 demonstrated the highest affinity towards both DiCDD and TCDD compared to previously analyzed pCYP1 enzymes (S3 Table; [12, 19]). The measured distances of DiCDD or TCDD from CYP1A2 heme’s oxygen atom were smaller than in CYP1A1 or CYP1B1, reflecting higher potential of being hydroxylated by the enzyme and suggesting a greater contribution of CYP1A2 to dioxin biodegradation. This assumption seems to be supported by the hepatic localization of pig CYP1A2 [21, 22, 34]. Some interesting differences were also noted in the behavior of the substrate channels after dioxin binding to different CYP1 molecules. Binding of DiCDD or TCDD to CYP1A2 resulted in a rapid closure of channel 2a, and channel S remained closed through the entire simulation time. In CYP1A1, the binding of TCDD resulted in channel S closure, however channel S remained open after binding DiCDD (S5 Fig; [12]). Similarly, both dioxins opened channel S after binding to CYP1B1 (S6 Fig; [19]). This seems particularly important since channel S is considered to be an exit channel from the enzyme’s active site. It cannot be excluded that the unavailability of channel S overcomes the impact of favorable affinity and the hydroxylating potential, and results in the ineffective biodegradation of TCDD by CYP1A2.

A few studies reported the formation of primary metabolites of TCDD in animal organisms as an indication of their biodegradation [25–27]. No research, however, demonstrated the occurrence of further stages of biodegradation or provided any reliable reasons for the lack of TCDD detoxification. In the current study, we analyzed molecular interactions between CYP1A2 and four dioxin metabolites. 3OH-DiCDD, a primary metabolite of DiCDD [11] and 8OH-TriCDD, a TCDD metabolite [14] showed a lower affinity to CYP1A2 than the respective original dioxin. However, the remaining TCDD metabolites *i*.*e*., 1OH-TCDD [26] and 2OH-TCDD [14] showed higher affinity to the pCYP1A2 active site than TCDD. Interestingly, the molecules of the two latter metabolites have more chlorine atoms than 8OH-TriCDD or 3OH-DiCDD. Numerous studies demonstrated that the increase in the binding affinity was accompanied by the greater number of chlorine atoms [28, 30]. To determine the probability of a hydroxylated product to be released from the active site, the distances between the iron atom of the heme and a particular dioxin metabolite were estimated. It should be emphasized that all calculated distances exceeded 5 Å (5.35–6.13 Å), strongly suggesting that the hydroxylated metabolites should not remain within the CYP1A2 active site [32]. The analysis of access channels revealed that channel 2c was opened only by 8OH-TriCDD. None of the metabolites bound to CYP1A2 promoted the opening of channel S. Contrary to the distances between the metabolites and the pCYP1A2 active site, the channel data suggest that the hydroxylated products are probably not capable of leaving the active site, which results in the blockage of the enzyme.

The active sites of pCYP1A2, CYP1A1 and CYP1B1formed by 12 canonical α-helices and 6 β-sheets were demonstrated to be deeply buried in the CYP molecule (the current study; [12, 19]). Similar to other CYP1 enzymes, the analysis of pCYP1A2 active site demonstrated the presence of a narrow and planar active site embedded in the enzyme molecule. The cavity volume of the CYP1A2 active site was close to that of CYP1A1 and considerably bigger than that of CYP1B1. The size and specific shape of the CYP1 active site affects hydroxylation of hydrophobic and planar compounds like PCDDs [11, 12]. The results of our previous study demonstrated, that smaller active site may result in the immobilization of a ligand, forcing a certain spatial orientation which may inhibit hydroxylation [19]. It is suggested, that enlarging the binding pocket through mutagenesis-derived specific changes in amino acids forming the helices of the enzyme’s active site, may ease TCDD biodegradation [9]. In summary, pCYP1A2 demonstrated the highest affinity towards both DiCDD and TCDD compared to other members of the pCYP1 family. Similar to the pCYP1A1 or pCYP1B1, all dioxin-pCYP1A2 complexes were found to be stabilized by hydrophobic interactions including π-stacking interactions. The distances between the heme oxygen atom and the dioxin carbon atom that is nearest to the oxygen indicated high hydroxylating potential of the enzyme–higher than those of other pCYP1 enzymes. Also, the distances from the heme iron atom did not suggest difficulties with metabolite egressing the active site. However, we also demonstrated that the binding of all dioxins resulted in a closure of access channel 2a, and the presence of metabolites did not promote opening of exit channel S. It is possible that such behavior of pCYP1A2 substrate channels overcomes the impact of the favorable affinity and hydroxylating potential of the enzyme, and results in the ineffective biodegradation of TCDD by CYP1A2.The presented data partially explain the molecular mechanisms underlying the slow and non-efficient degradation of TCDD by CYP enzymes. Although the results of the current study shed some light on the matter of CYP-mediated dioxin biodegradation, there are still more questions than answers.

## 4. Materials & methods

**Fig 8:**
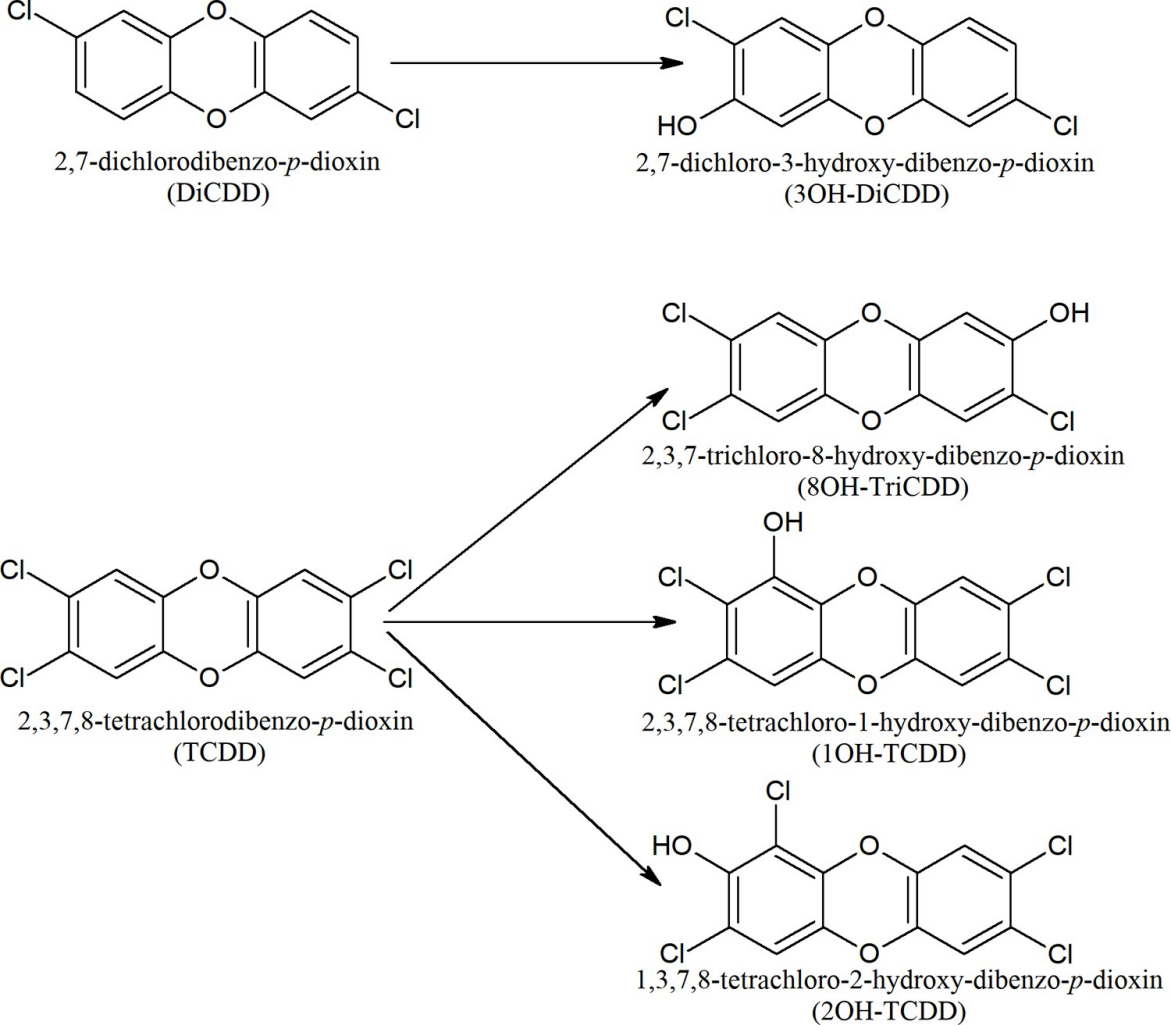
Chemical formulae of DiCDD and TCDD as well as their potential metabolites.

All stages of the study were performed for two dioxin congeners, characterized by distinctively different toxicity and susceptibility to biodegradation, i.e., DiCDD–less toxic and easily degraded, and TCDD–highly toxic and resistant to biodegradation. We also examined four dioxin metabolites: 3OH-DiCDD, 8OH-TriCDD, 1OH-TCDD and 2OH-TCDD. Chemical formulas of both dioxins and their metabolites are presented in Fig 8. Porcine *CYP1A2* cDNA sequence was established experimentally by next generation sequencing (NGS; [6]). In our previous experiment, total RNA was isolated from porcine liver and tested for concentration, quality and integrity. Next, cDNA strands were synthesized and DNA libraries were amplified and quantified. Finally, libraries were sequenced on llumina HiSeq2500 high throughput sequencing instrument (OpenExome, Poland) with 100 paired-end (PE) sequencing [6]. The porcine *CYP1A2* (pCYP1A2) sequence was localized in the transcriptome, trimmed and submitted to GenBank under the following accession number: AIY35109.1. The nucleotide (nt) sequence was then translated to amino acid (aa) sequence.

### 4.1. Homology modeling of the pCYP1A2 catalytic domain

The amino acid sequence of pCYP1A2 protein was used to perform homology modeling of the enzyme catalytic domain (aa 34–516). The crystalline structure of human CYP1A2 (PDB ID: 2HI4) was chosen as a template. The sequence alignment between pCYP1A2 and the template was performed with the use of MUSCLE software [35]. A tertiary structure of pCYP1A2 was generated using Modeller 9v14 software [36]. Models with the lowest Discrete Optimized Protein Energy (DOPE) score were selected for further analysis. The reliability of the constructed models was evaluated with the use of PROCHECK, ProSA-web and VERIFY3D [37–40]. The model with the best validation scores was selected to be further analyzed. The cavity volume of the pCYP1A2 active site was calculated using VOIDOO software [41]. The analysis was carried out with the probe-occupied algorithm. The probe radius and primary grid spacing were set to 1.4 Å and 0.33 Å, respectively. Other calculation parameters were set to default values.

### 4.2. Molecular docking of dioxins to the pCYP1A2 active site

Spatial structures of the two selected dioxin congeners (DiCDD and TCDD) and their metabolites (8OH-TriCDD, 3OH-DiCDD,2OH-TCDD,1OH-TCDD) were obtained from ZINC database [42]. Prior to dioxin docking, hydrogen atoms and charges were added to the modeled structure of pCYP1A2 with the use of MGL Tools 1.5.4 [43]. The parameters of the ferric heme were applied [44]. The molecular docking was performed using AutoDock Vina 1.1.2 program [45]. The grid box was constructed around the active site of pCYP1A2 protein with X, Y, Z parameters set to 15, 15 and 15 Å. The docking analysis was performed with exhaustiveness of 32. Other parameters were set to default values. The program generated 20 results reflecting the best spatial positions of a dioxin molecule within the pCYP1A2 active site. For each dioxin, the complex with the lowest energetic binding score was chosen as the most energetically favorable, and was used to perform the subsequent molecular dynamics (MD) simulation. To confirm the high reliability of the constructed homology model of porcine CYP1A2, a corresponding analysis was performed for the crystalline structure of human CYP1A2 (PDB ID: 2HI4).

### 4.3. Molecular dynamics simulation of the PCDD-pCYP1A2 complexes

Molecular dynamics simulations were carried out with GROMACS 4.6.7 software using ffamber99sb force field [46, 47]. The heme parameters were applied following [44], using the heme Compound I intermediate (CpdI). To obtain force fields of the examined dioxins, the spatial geometry of their structure was optimized with the use of Gaussian 09 software, applying B3LYP/6-31G(d) level of theory [48]. Afterwards, the partial charges were obtained by the restrained electrostatic potential fitting technique (RESP) based on electrostatic potentials (Gaussian 09 software) and applying Hartree-Fock (HF) SCF/6-31G(d) level of theory [49]. For MD simulation, a rhombic dodecahedron box of explicit TIP3P water molecules was constructed around the examined complexes in a 10 Å distance from every peripheral residue. Sodium and chlorine ions were added to neutralize the system [50].

Each PCDD-pCYP1A2 complex was relaxed using a harmonic constant of 1000 J·mol^−1^·nm^−1^ with restraints on protein heavy atoms. The energy minimization was conducted with the steepest-descent algorithm through 5000 steps. The system was first heated from 0 to 300 K for 500 ps under NVT (constant number of particles, volume and temperature) conditions. Next, the system was equilibrated under NPT (constant number of particles, pressure and temperature) conditions for 1 ns. The relaxed dioxin-pCYP1A2 complexes underwent a 300 ns MD simulation under NPT conditions. Periodic boundary conditions and 12 Å cut-off for non-bonded van der Waals (vdW) interactions were applied. Particle Mesh Ewald (PME) algorithm was used to calculate the long-range electrostatic interactions between atoms. All bonds involving hydrogen atoms were constrained by LINCS algorithm [51]. The time step for MD simulation was set to 2 fs. In addition, MD simulation of the substrate-free form (APO) of porcine CYP1A2 was performed to exclude the effect of substrate binding on protein structure.

### 4.4. Binding free energy of the PCDD-pCYP1A2 complexes

In the present study we used two different methods to predict binding free energy, i.e., absolute binding free energy (ABF) and thermodynamic integration (TI). ABF was employed to estimate the binding affinity of dioxin molecules to the pCYP1A2 active site [52]. TI was used to determine the impact of selected aa residues on a dioxin stabilization within the pCYP1A2 active site [53].

#### 4.4.1. Absolute binding free energy

Calculations of ABF for the PCDD-CYP1A2 complexes were performed with the use of a non-physical thermodynamic cycle depicted in S7 Fig. Two systems were analyzed in the cycle in the presence of water molecules: 1/ PCDD molecule was bound to the active site of pCYP1A2 and 2/ unbound PCDD molecule. To estimate the ABF between a PCDD and the pCYP1A2 active site, the ligand van der Waals and electrostatic interactions were decoupled (Δλ = 0.05) and annihilated (Δλ = 0.1), respectively (S7 Fig, A→B). Next, the relative position and orientation of the PCDD bound to the pCYP1A2 were restrained by harmonic potential using a harmonic constant (4184.0J·mol^−1^·nm^−1^for distances; 41.84 J·mol^−1^·nm^−1^for angles). These restraints were executed by twelve non-uniformly distributed λ states ranged from 0 to 1 (0.000, 0.010, 0.025, 0.050, 0.075, 0.100, 0.150, 0.200, 0.300, 0.500, 0.750, 1.000). The relaxation of the systems for each λ-state was performed as described in Section 2.3 (*Molecular dynamics simulation of the PCDD-pCYP1A2 complexes*) with the use of GROMACS 5.1.4. Calculations of ABF were performed during 40 ns with a 2 fs time-step. A soft-core potential was employed to improve the transformation of van der Waals interactions. Particle Mesh Ewald (PME) algorithm was used to calculate the long-range electrostatic interactions between atoms. All bonds involving hydrogen atoms were constrained by P-LINCS algorithm. The g_bar tool (GROMACS package) implementing the Bennet’s acceptance ratio method (BAR) was used to estimate the ABF of PCDD bound to the pCYP1A2 active site. To reach an equilibrium, the first 10 ns of each simulation was discarded.

#### 4.4.2. Thermodynamic integration

To determine the impact of selected aa residues on a dioxin stabilization within the pCYP1A2 active site, the residues were mutated during TI calculation. Calculations of binding free energy for PCDD-pCYP1A2 complexes were performed with the use of the thermodynamic cycle depicted in S2 Fig. The residues important for a dioxin stabilization within the enzyme active site (wild-type; WT) were designated for mutation (Mut) following the MD simulations. Briefly, the difference in binding free energy between aPCDD-pCYP1A2_WT_ complex and aPCDD-pCYP1A2_MUT_ complex was estimated on the basis of an alchemical transformation with the use of λ parameter coupling. The total energy of each state is described by its Hamiltonian (H) i.e., H0 and H1 for thePCDD-pCYP1A2_WT_ complex and thePCDD-pCYP1A2_MUT_ complex, respectively. The transformation between the two states is described by adding the λ parameter to the H. The intermediate values of the λ parameter represent intermediate states of the transformation. The integration of the free energy values along a continuous path connecting the initial (H0) and final (H1) state was used to estimate the difference in binding free energy as follows:

$${\Delta G}_{binding}(A\to B)=\int_{0}^{1}{\langle \frac{\delta H(q,p,\lambda )}{\delta \lambda }\rangle }_{\lambda }d\lambda$$

where, q stands for the atomic position, p stands for the linear momentum, and the angular bracket stands for a Boltzmann-weighted ensemble average at a particular λ value. The difference in the relative binding free energy (ΔΔG) between the PCDD-pCYP1A2_WT_ complex and the PCDD-pCYP1A2_MUT_ complex can be calculated with the use of a thermodynamic cycle (S8 Fig). Based on the cycle, the ΔΔG can be calculated according to the following equation:

$${\Delta \Delta G}_{\left(WT\to Mut\right)}={\Delta G}_{binding}(WT)-{\Delta G}_{binding}(Mut)={\Delta G}_{bound(WT\to Mut)}+{\Delta G}_{unbound(WT\to Mut)}$$

where, ${\Delta G}_{bound(WT\to Mut)}$ stands for alchemical transformation of the ligand-bound form ofwild-type pCYP1A2 to the mutated pCYP1A2, ${\Delta G}_{unbound(WT\to Mut)}$ represents alchemical transformation of the ligand free-form of wild-type the pCYP1A2 to the mutated pCYP1A2 protein.

For calculation of ΔΔG, the wild-type of pCYP1A2 was used as a reference. Two systems were analyzed in the cycle in the presence of water molecules: 1/ a PCDD molecule bound to the active site of pCYP1A2 and 2/ ligand-free form of the pCYP1A2 protein; thirty λ-states were established in each system. The relaxation of the complexes for each λ-state was performed as described in Section 2.3 (*Molecular dynamics simulation of the PCDD-pCYP1A2 complexes*). The free energy calculation was performed during 30 ns with the use of Hamiltonian replica exchange dynamics with a 2 fs time-step. According to the Gibbs sampling scheme, 3 million swaps between any state pair were performed for every 1000 time steps. The g_bar tool (GROMACS package) implementing the Bennet’s acceptance ratio method (BAR) was used to estimate the difference in relative binding free energy (ΔΔG) between wild-type and mutant pCYP1A2 protein bound TCDD or DiCDD within the enzyme active site. To reach equilibrium, the first 10 ns of each simulation was discarded. These differences reflect the importance of particular residues in dioxin stabilization. The obtained positive/negative values denote that the interactions between the substituted residues and a dioxin were weaker/stronger, respectively, than those of the corresponding “wild type” residues.

### 4.5. Access channels analysis

Access channels of pCYP1A2 molecule were analyzed using CAVER 3.0.1 software [54]. The snapshots of MD simulation trajectories of the dioxin-pCYP1A2 complexes as well as the unbound CYP1A2 were extracted at every 50 ps from 100 to 300 ns. The probe radius and clustering threshold of each channel were set to 1.2 Å and 3.5 Å, respectively. Default settings for other parameters were used throughout the calculations. The beginning of each channel was localized 3 Å above the iron atom of the heme molecule. The tunnels were visualized with the use of PyMOL software [55]. To extend the knowledge concerning a potential ability of porcine CYP1A2 enzyme to hydroxylate a dioxin molecule, a corresponding analysis was performed for dioxin metabolites bound within the enzyme active site.

## Supporting information

S1 Table The protein sequence identity between the CYP1A2 of the pig and other species. (XLSX)

S2 Table The average root-mean-square deviation (RMSD) values for the examined dioxin-CYP1A2 complexes during MD simulations. (DOCX)

S3 Table The absolute binding free energy (kcal·mol^-1^) calculated for the PCDD-CYP1 complexes. (DOCX)

S1 Fig The amino acid sequence alignment between catalytic domains of porcine CYP1A2 and human CYP1A2 templates. Yellow background and red or black letters indicate that the sequence homology is high, medium or low, respectively. Spirals represent α-helices, arrows represent β-strands, blue stars indicate amino acids involved in the ligand binding to the enzyme active site.
(TIF)

S2 Fig Ramachandran plot for the generated homology-based model of pCYP1A2. (TIF)

S3 Fig The visualization of a dioxin position in the active site of pCYP1A2 after molecular docking. Side chains of pCYP1A2 amino acids interacting with each of the examined dioxin are depicted in yellow; heme is black; DiCDD is beige, TCDD is lavender, 3OH-DiCDD is green, 8OH-TriCDD is pale pink, 1OH-TCDD is pale willow-green and 2OH-TCDD as orange.
(TIF)

S4 Fig Time-dependent changes in the distance between the hCYP1A2 heme oxygen and the dioxin carbon atom that is nearest to the oxygen (a, b) and in the angle between the hCYP1A2 heme iron, oxygen and the dioxin carbon atom that is nearest to the oxygen (c, d).
(TIF)

S5 Fig Dioxin-induced changes in the availability of the access channels identified within the pCYP1A1 molecule; APO–substrate free form of pCYP1A1. (TIF)

S6 Fig Dioxin-induced changes in the availability of the access channels identified within the pCYP1B1 molecule; APO–substrate free form of pCYP1B1. (TIF)

S7 FigThe thermodynamic cycle used to estimate the absolute binding free energy of a dioxin bound to pCYP1A2.Ligand of the pCYP1A2 is shown either as bound to the active site of the enzyme (right panel) or unbound (left panel) in the environment of water molecules (bluish background). A) The PCDD molecule (blue), fully able to interact with water molecules is alchemically transformed into B) non-interacting molecule (white). This transformation $\left({\Delta G}_{elec+vdw}^{solv}\right)$ was conducted with a series of simulations in which electrostatic (*elec*) and van der Waals (*vdw*) interactions between the ligand and water molecules are scaled to zero. C) Next, a non-interacting PCDD molecule was restrained (red pin). This transformation $\left({\Delta G}_{rest}^{solv}\right)$ led to the state which is equivalent to D) non-interacting PCDD molecule restrained within the pCYP1A2 active site. E) Then, the *elec* and *vdw* interactions of the restrained PCDD molecule bound to the pCYP1A2 active site were gradually reinstated (${\Delta G}_{elec+vdw}^{prot}$).F) Finally, the positional restraints of PCDD molecule bound to the pCYP1A2 active site were removed, and the unrestrained dioxin is fully able to interact with the enzyme (${\Delta G}_{rest}^{prot}$).(TIF)

S8 Fig The thermodynamic cycle used to determine the impact of particular amino acid residues on dioxin stabilization within the pCYP1A2 active site. (TIF)
