# Supplementary material for: The involvement of CYP1A2 in biodegradation of dioxins in pigs
Source: PLoS One. 2022 May 26;17(5):e0267162. doi: 10.1371/journal.pone.0267162 (PMC9135293; doi:10.1371/journal.pone.0267162)
Supplement: S2 Table — (DOCX) [file pone.0267162.s002.docx]

S2 Table

| **Complex** | **RMSD (Å)** |
| --- | --- |
| APO^a^ | 2.04±0.15 |
| DiCDD-pCYP1A2 | 1.98±0.16 |
| TCDD-pCYP1A2 | 2.02±0.18 |
| 3OH-DiCDD-pCYP1A2 | 2.04±0.12 |
| 8OH-TriCDD-pCYP1A2 | 1.91±0.15 |
| 1OH-TCDD-pCYP1A2 | 1.97±0.21 |
| 2OH-TCDD-pCYP1A2 | 1.90±0.12 |

^a^Apo is a substrate-free form of pCYP1A2 protein
