# Supplementary material for: The involvement of CYP1A2 in biodegradation of dioxins in pigs
Source: PLoS One. 2022 May 26;17(5):e0267162. doi: 10.1371/journal.pone.0267162 (PMC9135293; doi:10.1371/journal.pone.0267162)
Supplement: S3 Table — (DOCX) [file pone.0267162.s003.docx]

S3 Table

| **Energy component** | **DiCDD** | | | **TCDD** | | |
| --- | --- | --- | --- | --- | --- | --- |
|  | **pCYP1A1** | **pCYP1A2** | **pCYP1B1** | **pCYP1A1** | **pCYP1A2** | **pCYP1B1** |
| ${\boldsymbol{\Delta}\boldsymbol{G}}_{\boldsymbol{elec}\boldsymbol{+}\boldsymbol{vdw}\boldsymbol{+}\boldsymbol{rest}}^{\boldsymbol{prot}}$ | -16.300±0.073 | -22.369±0.022 | -19.325±0.082 | -21.809±0.063 | -23.335±0.013 | -20.141±0.071 |
| ${\boldsymbol{\Delta}\boldsymbol{G}}_{\boldsymbol{elec}\boldsymbol{+}\boldsymbol{vdw}}^{\boldsymbol{solv}}$ | 2.611±0.014 | 2.611±0.014 | 2.611±0.014 | 2.497±0.016 | 2.497±0.016 | 2.497±0.016 |
| ${\boldsymbol{\Delta}\boldsymbol{G}}_{\boldsymbol{rest}}^{\boldsymbol{solv}}$ | 6.906 | 6.980 | 7.092 | 7.055 | 6.894 | 7.167 |
| ${\boldsymbol{\Delta}\boldsymbol{G}}_{\boldsymbol{binding}}^{\boldsymbol{0}}$ | **-6.783±0.074** | **-12.778±0.026** | **-9.622±0.083** | **-12.145±0.065** | **-13.671±0.02** | **-10.477±0.073** |

${\Delta G}_{elec+vdw+rest}^{prot}$– ligand decoupling from complex; ${\Delta G}_{elec+vdw}^{solv}$ – ligand decoupling from solution; ${\Delta G}_{rest}^{solv}$– ligand restraints added to decoupled ligand; ${\Delta G}_{binding}^{0}$– absolute binding free energy, ${\Delta G}_{binding}^{o}={\Delta G}_{elec+vdw+rest}^{prot}+{\Delta G}_{elec+vdw}^{solv}+{\Delta G}_{rest}^{solv}$

DiCDD – 2,7-dichlorodibenzo-*p*-dioxin, TCDD – 2,3,7,8-tetrachlorodibenzo-*p*-dioxin
