# Supplementary figures and images for: The involvement of CYP1A2 in biodegradation of dioxins in pigs
Source: PLoS One. 2022 May 26;17(5):e0267162. doi: 10.1371/journal.pone.0267162 (PMC9135293; doi:10.1371/journal.pone.0267162)

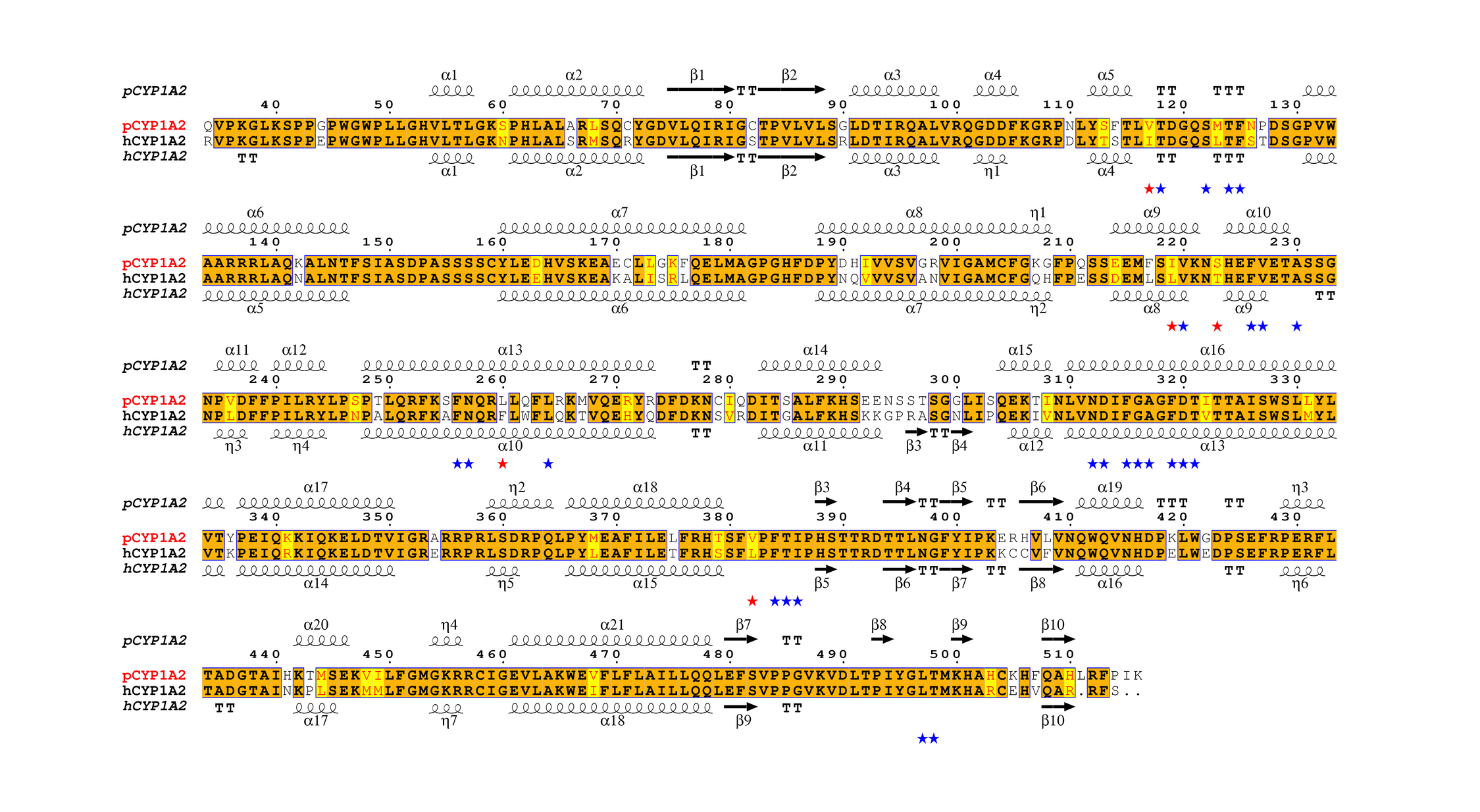

Supplement: S1 Fig — Yellow background and red or black letters indicate that the sequence homology is high, medium or low, respectively. Spirals represent α-helices, arrows represent β-strands, blue stars indicate amino acids involved in the ligand binding to the enzyme active site. (TIF) [file pone.0267162.s004.tif]

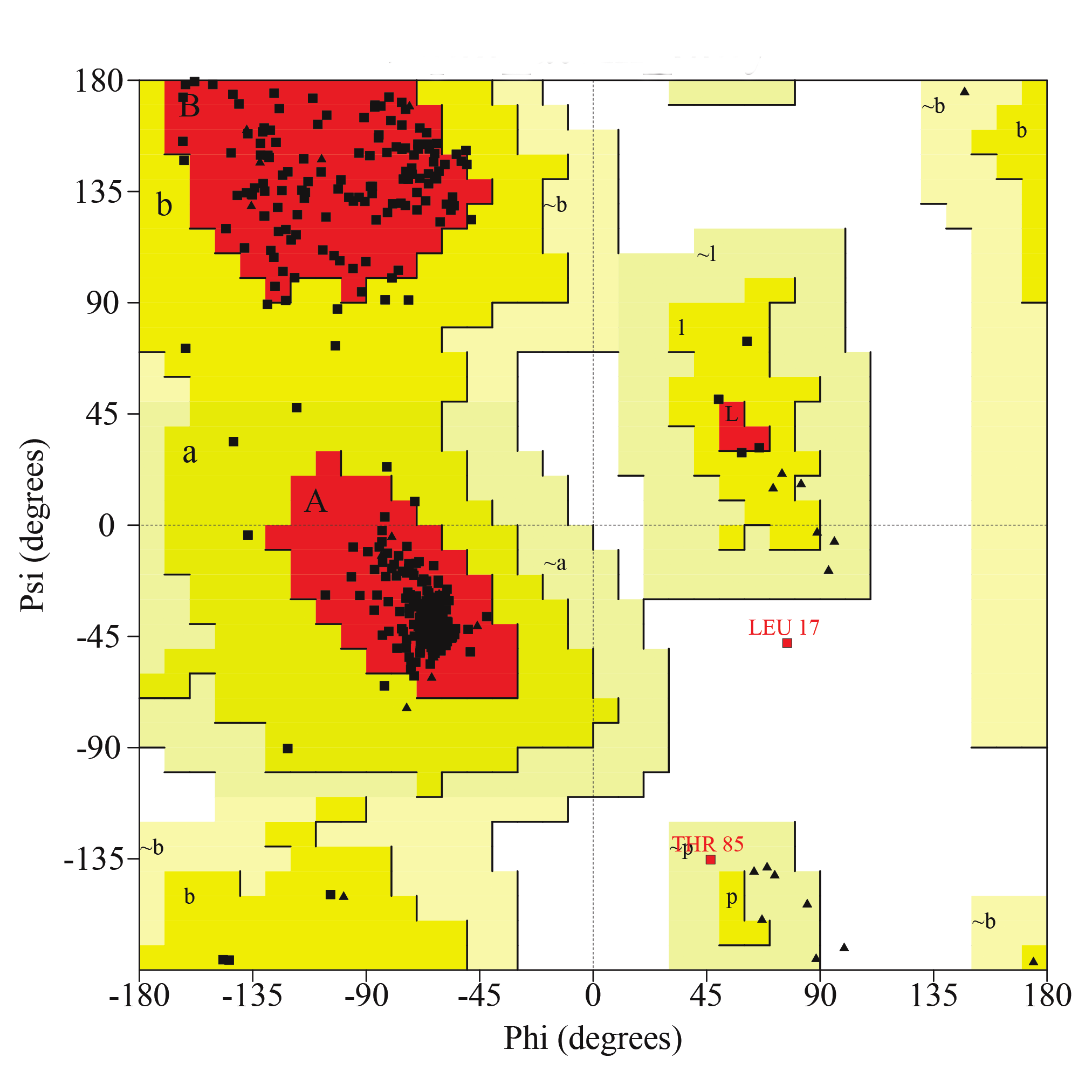

Supplement: S2 Fig — (TIF) [file pone.0267162.s005.tif]

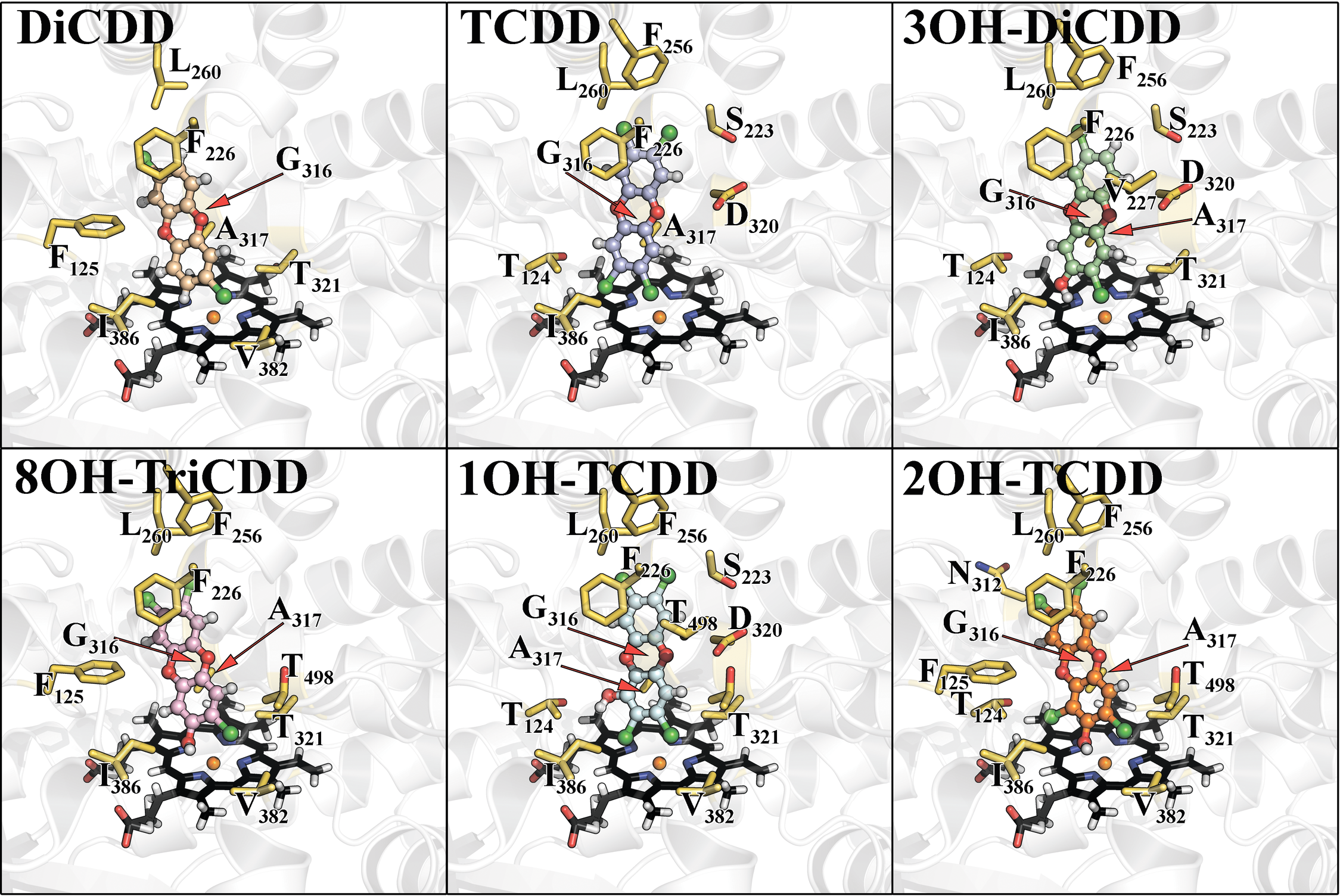

Supplement: S3 Fig — Side chains of pCYP1A2 amino acids interacting with each of the examined dioxin are depicted in yellow; heme is black; DiCDD is beige, TCDD is lavender, 3OH-DiCDD is green, 8OH-TriCDD is pale pink, 1OH-TCDD is pale willow-green and 2OH-TCDD as orange. (TIF) [file pone.0267162.s006.tif]

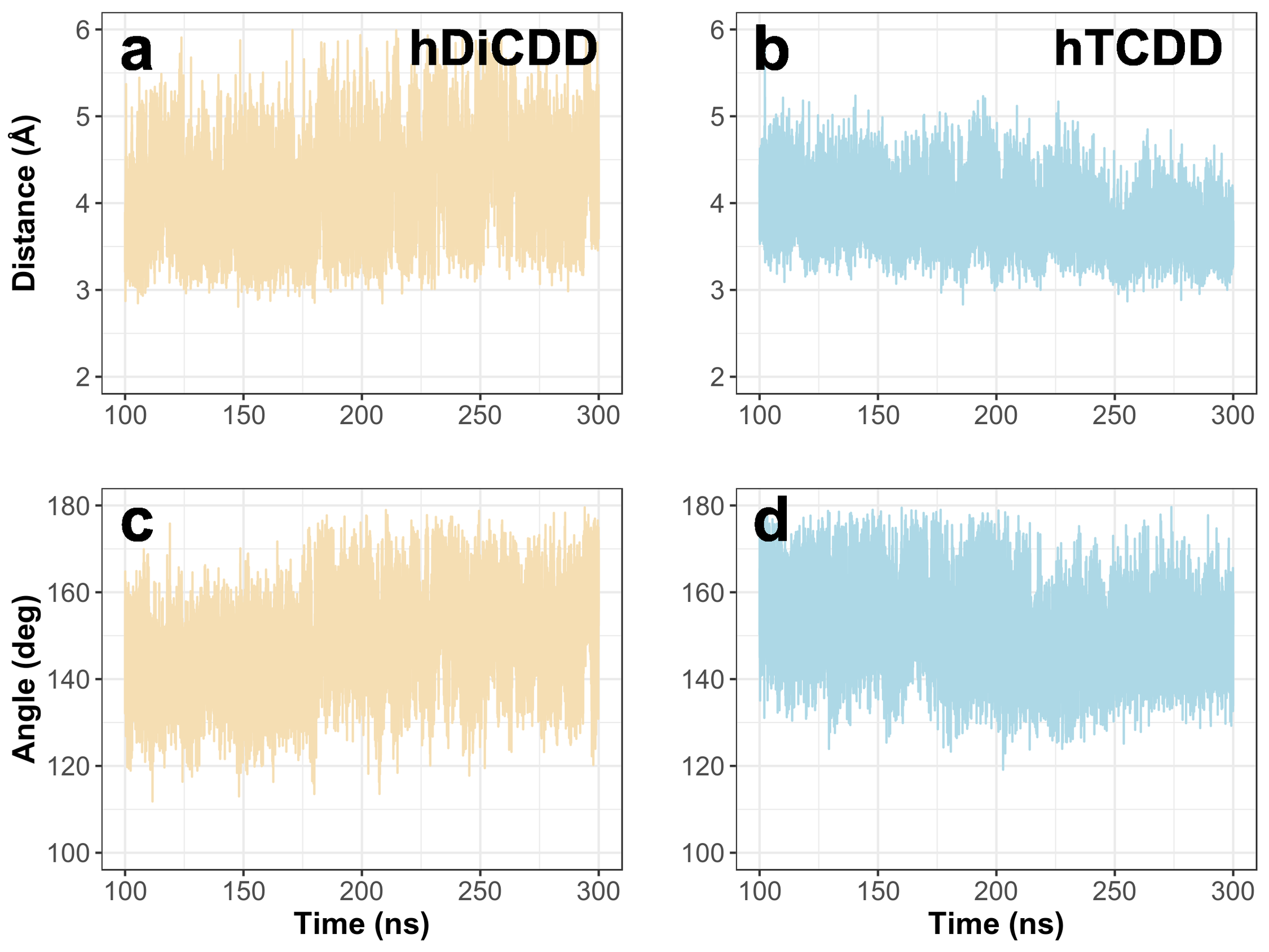

Supplement: S4 Fig — Time-dependent changes in the distance between the hCYP1A2 heme oxygen and the dioxin carbon atom that is nearest to the oxygen (a, b) and in the angle between the hCYP1A2 heme iron, oxygen and the dioxin carbon atom that is nearest to the oxygen (c, d). (TIF) [file pone.0267162.s007.tif]

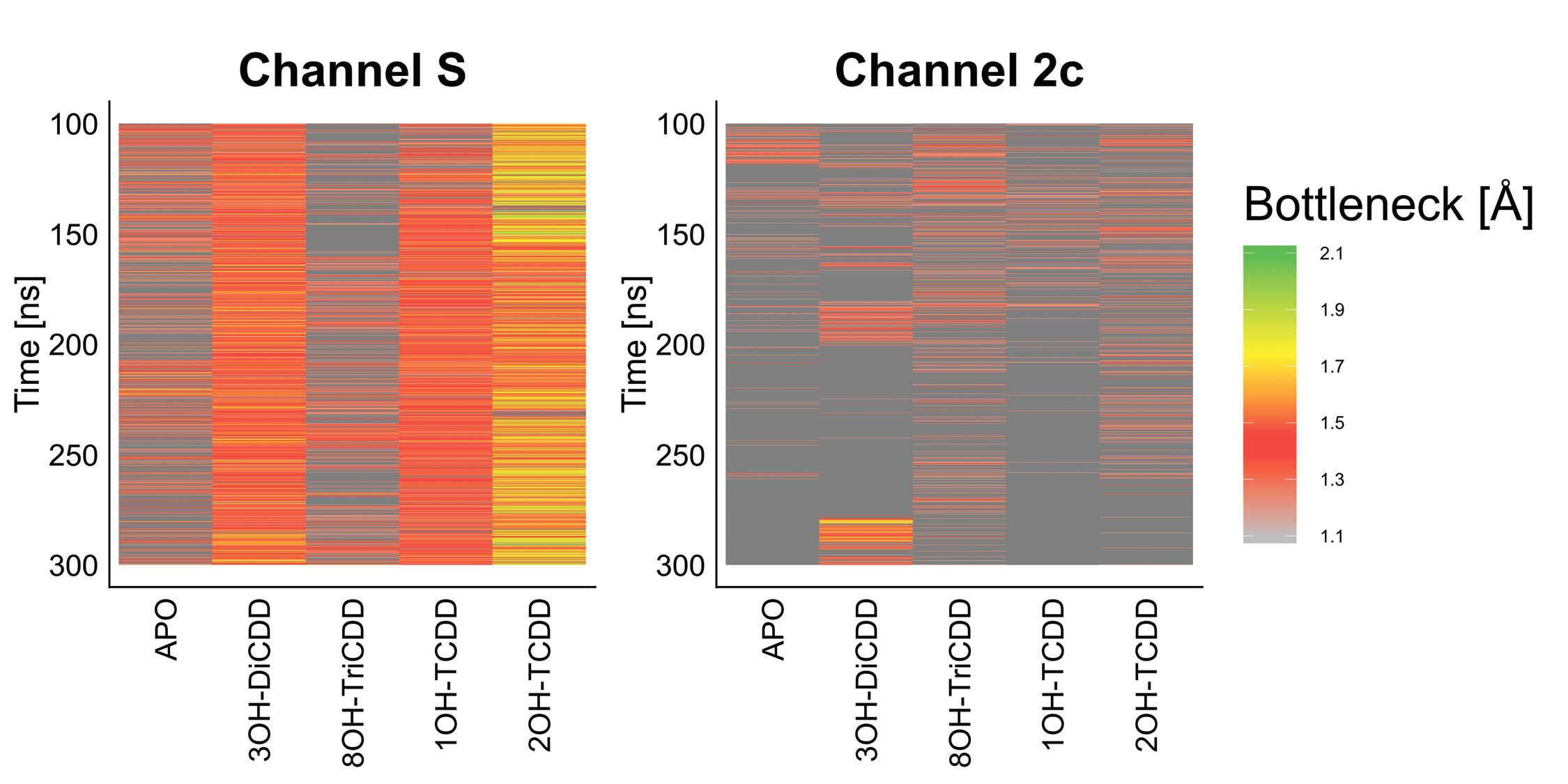

Supplement: S5 Fig — (TIF) [file pone.0267162.s008.tif]

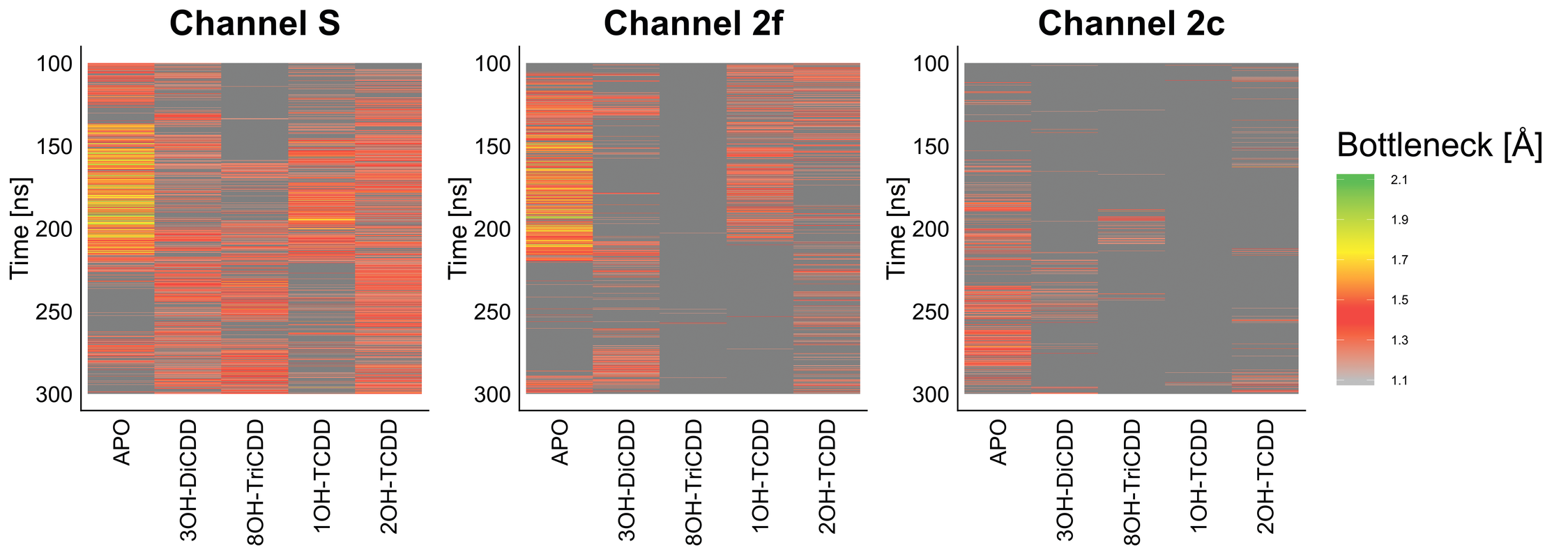

Supplement: S6 Fig — (TIF) [file pone.0267162.s009.tif]

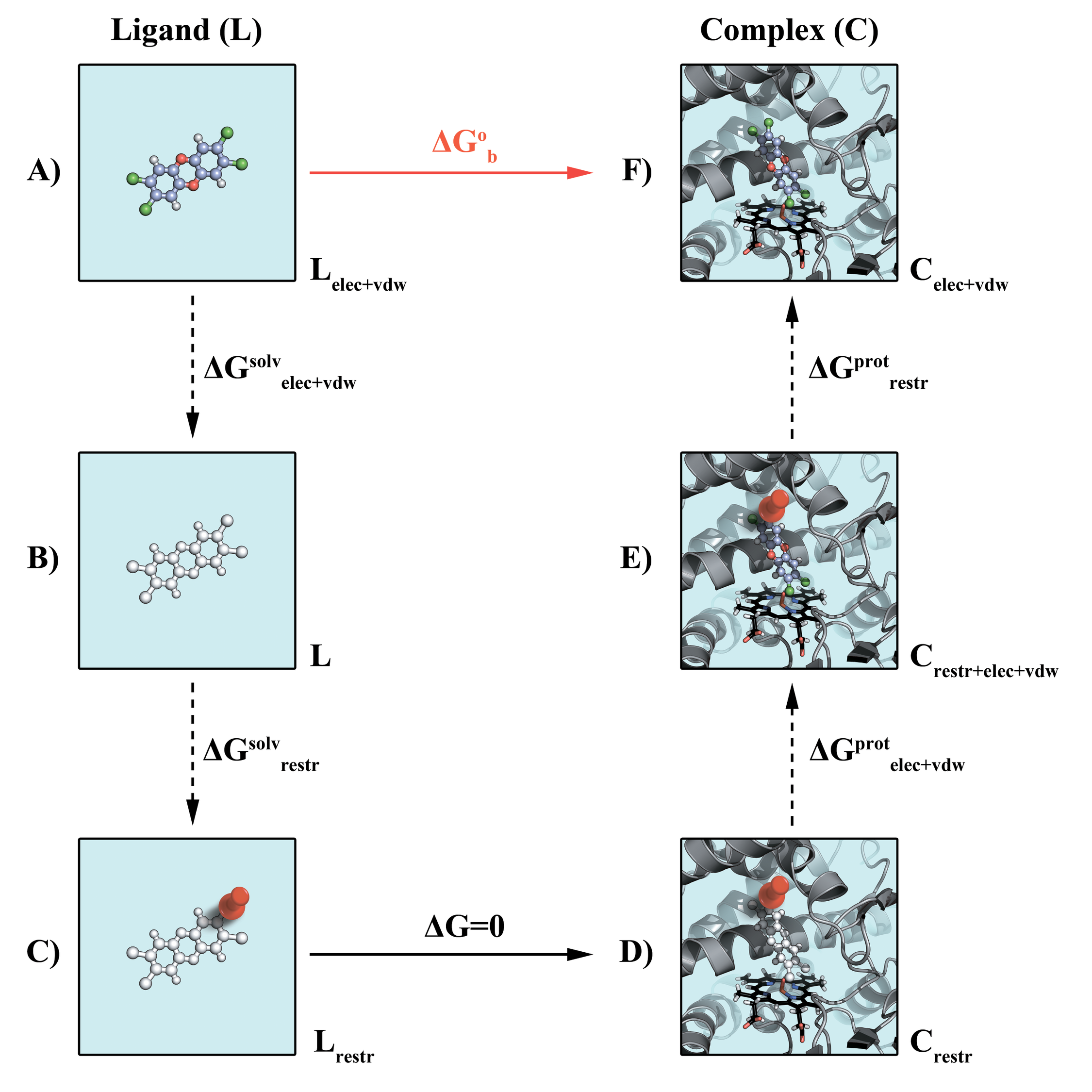

Supplement: S7 Fig — Ligand of the pCYP1A2 is shown either as bound to the active site of the enzyme (right panel) or unbound (left panel) in the environment of water molecules (bluish background). A) The PCDD molecule (blue), fully able to interact with water molecules is alchemically transformed into B) non-interacting molecule (white). This transformation (ΔGelec+vdwsolv) was conducted with a series of simulations in which electrostatic (elec) and van der Waals (vdw) interactions between the ligand and water molecules are scaled to zero. C) Next, a non-interacting PCDD molecule was restrained (red pin). This transformation (ΔGrestsolv) led to the state which is equivalent to D) non-interacting PCDD molecule restrained within the pCYP1A2 active site. E) Then, the elec and vdw interactions of the restrained PCDD molecule bound to the pCYP1A2 active site were gradually reinstated (ΔGelec+vdwprot).F) Finally, the positional restraints of PCDD molecule bound to the pCYP1A2 active site were removed, and the unrestrained dioxin is fully able to interact with the enzyme (ΔGrestprot). (TIF) [file pone.0267162.s010.tif]

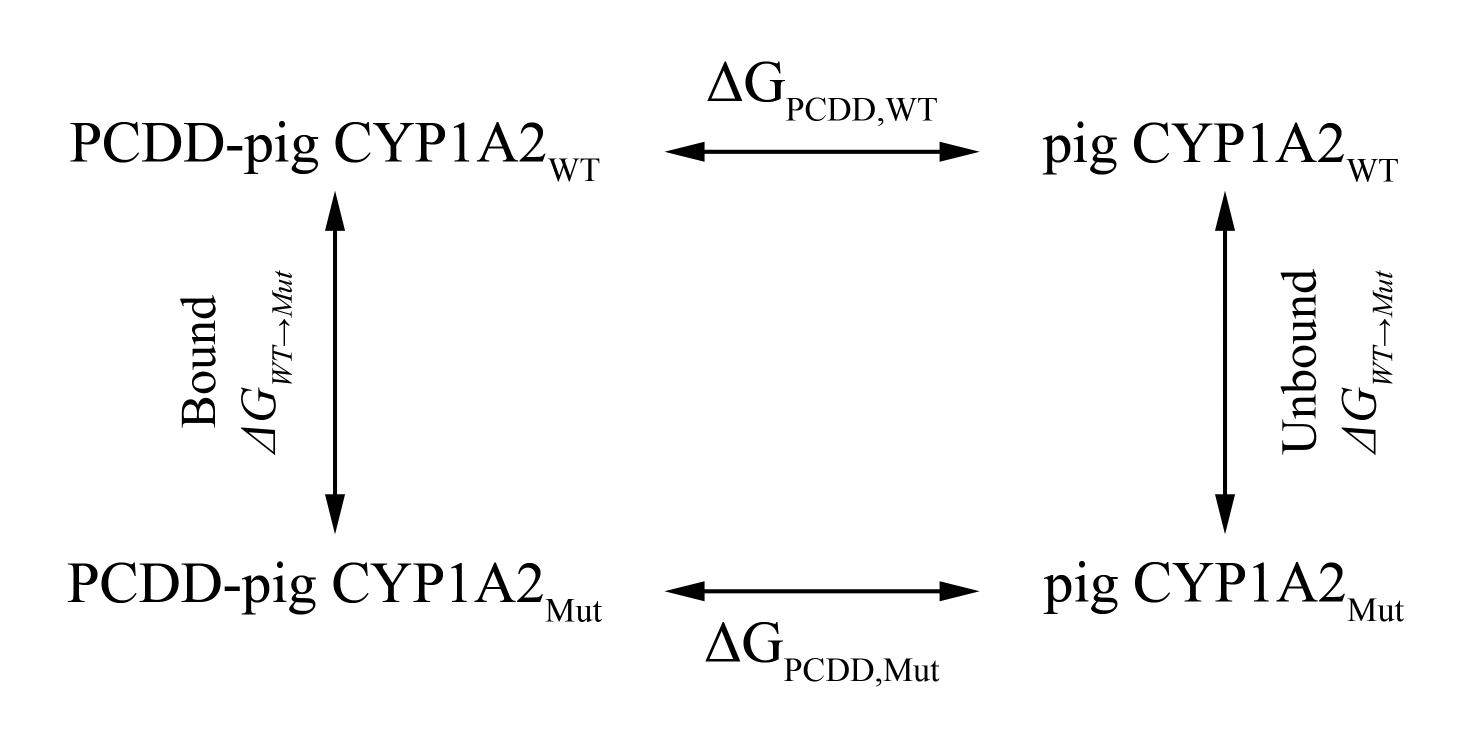

Supplement: S8 Fig — (TIF) [file pone.0267162.s011.tif]
